# Antiviral effects and mechanism of Ma-Xing-Shi-Gan-San on porcine reproductive and respiratory syndrome virus

**DOI:** 10.3389/fmicb.2025.1539094

**Published:** 2025-04-29

**Authors:** Miao Zhang, Jiankun Huang, Qingan Chi, Xuhua Ran, Xiaobo Wen

**Affiliations:** ^1^School of Tropical Agriculture and Forestry, Hainan University, Haikou, Hainan, China; ^2^Hainan Animal Disease Prevention and Control Center, Haikou, Hainan, China

**Keywords:** porcine reproductive and respiratory syndrome virus, traditional Chinese medicine, Ma-Xing-Shi-Gan-San, network pharmacology, molecular docking

## Abstract

**Background:**

Currently, vaccination has consistently posed challenges in preventing the Porcine reproductive and respiratory syndrome virus (PRRSV), so there is an urgent need for effective controlling strategies. Ma-Xing-Shi-Gan-San (MXSGS), a traditional Chinese medicine (TCM) formula used for pulmonary diseases and respiratory disorders, has proven effective in treating H1N1 and COVID-19. Herein, we evaluated whether MXSGS exhibits potent antiviral activity against PRRSV.

**Methods:**

First, a PRRSV-infected Marc-145 cell model was established. Reverse transcription-quantitative polymerase chain reaction (RT-qPCR) and the tissue culture infective dose (TCID₅₀) assay were performed to assess the inhibitory effects of MXSGS on PRRSV during different administration stages. Network pharmacology was then employed to identify key active ingredients and core potential targets of MXSGS against PRRSV. In addition, gene ontology (GO) and Kyoto Encyclopedia of Genes and Genomes (KEGG) analyses were conducted to elucidate the antiviral signaling pathways modulated by MXSGS. Lastly, candidate ingredients and targets were validated by molecular docking analysis.

**Results:**

MXSGS significantly inhibited PRRSV through prophylactic and therapeutic administration and suppressed multiple phases of the viral life cycle, including attachment, internalization, replication, and release. In network pharmacology results, 82 active ingredients and 118 therapeutic targets related to MXSGS and PRRSV were identified. Among them, Calycosin, Odoratin, Glyzaglabrin, 7,2′,4′-trihydroxy-5-methoxy-3-arylcoumarin, and Eriodictyol were selected as key active ingredients. ALB, PPARG, CASP3, STAT3, TGFB1, JAK2, TLR4, PRKACA, and PRKACB were screened as potential core targets. Furthermore, pathway and functional enrichment analysis revealed that the impact of MXSGS on PRRSV mainly involved Toll-like receptor signaling pathway, typical NF-κB signaling, positive regulation of interleukin-6 production, Th17 cell differentiation, inflammatory response, and viral defense response. Lastly, molecular docking analysis indicated an excellent binding affinity between the core potential targets and key active ingredients, with all binding energies < −6.0 kcal/mol.

**Conclusion:**

*In vitro* experiments indicated that MXSGS exhibited considerable anti-PRRSV activity. Using network pharmacology and molecular docking approaches, five key active ingredients and six core potential targets were identified, underscoring MXSGS as a promising pharmaceutical agent for controlling PRRSV.

## Graphical abstract

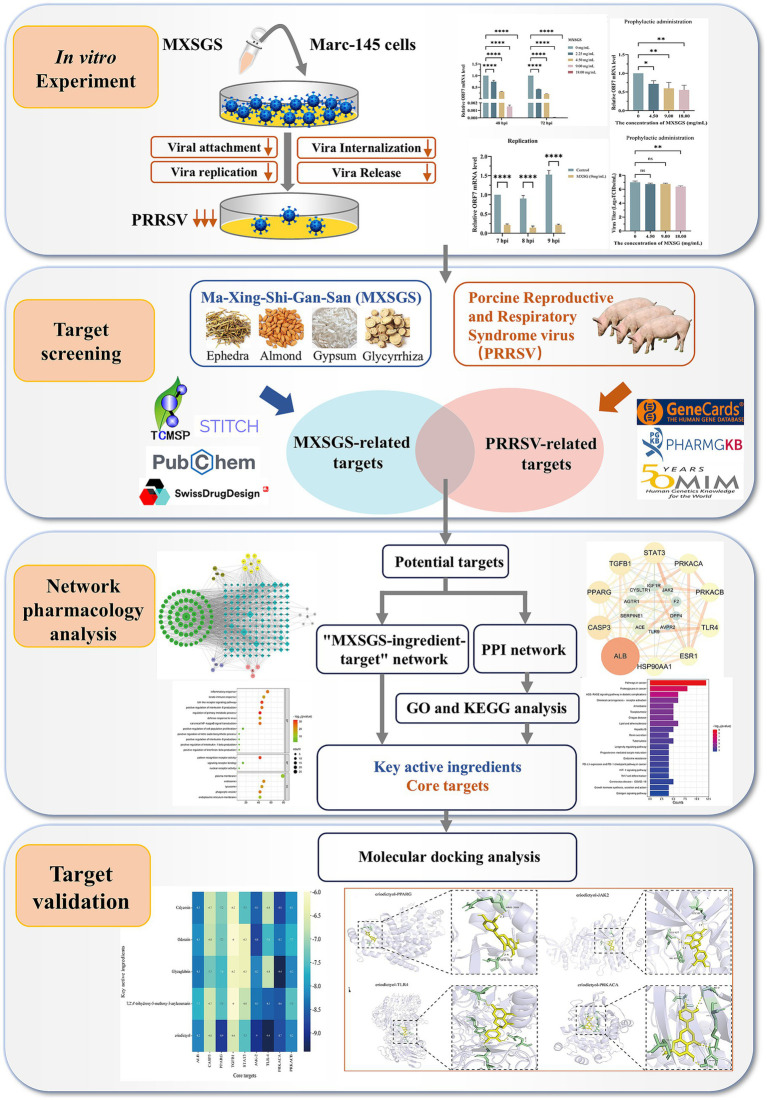

## 1 Introduction

Porcine reproductive and respiratory syndrome (PRRS) is a globally endemic viral disease in swine caused by lung infections, manifesting as reproductive failure and severe respiratory syndrome with high piglet mortality (Li et al., 2022; Cui et al., 2024). The causative agent of this disease is PRRS virus (PRRSV), an enveloped, positive-strand RNA virus belonging to the order Nidovirales and the family *Arteriviridae* (Li C. et al., 2024). Based on genetic diversity and geographic distribution, PRRSV can be broadly categorized into two species: *Betaarterivirus suid 1* and *Betaarterivirus suid 2*, which share approximately 60% nucleotide sequence homology (Sun et al., 2024).

Currently, vaccination is the primary strategy for preventing PRRSV infection (Du et al., 2017). However, inactivated vaccines have proven challenging in eliciting sufficient neutralizing antibodies, and attenuated vaccines were difficult to induce sterilizing immunity against various strains (Li J. et al., 2024). Although some alternative antiviral agents like Ribavirin, type I IFN, etc., revealed the ability against PRRSV, are prone to developing potential resistance mutation issues, ultimately resulting in successful PRRSV infections (Sang et al., 2011; Khatun et al., 2016). Traditional Chinese Medicines (TCMs) and their natural compounds are increasingly recognized as a viable approach for managing PRRSV due to their affordability, broad-spectrum antiviral abilities, low drug resistance, reduced drug resistance, and minimal adverse effects (Bello-Onaghise et al., 2020; Huang et al., 2021; Liu et al., 2025). For example, Fu-Zheng-Jie-Du-San (FZJDS), a compound Chinese herb, could inhibit PRRSV proliferation *in vitro* by targeting the PI3K/Akt signaling pathway (Chang et al., 2024). (−)-Epigallocatechin-3-gallate (EGCG), the most abundant catechin in green tea, prevented PRRSV attachment to Marc-145 cells by downregulating the expression of key receptors such as CD163, MYH9, and HS (Ge et al., 2018).

Ma-Xing-Shi-Gan-San (MXSGS), a multi-component compound of TCM developed by the Typhoid and Fever School of Pharmaceutical Biology formulation, consists of *Ephedra sinica* (Chinese name: Mahuang), Semen armeniacae amarum (Chinese name: Kuxingren), Gypsum Fibrosum (Chinese name: Shigao) and Glycyrrhiza uralensis (Chinese name: Gancao) (Ye et al., 2023). MXSGS is mainly used to treat fever and lung diseases, exhibiting antiviral and anti-inflammation activity in treatment of the COVID-19 (Li et al., 2021). Specifically, PRRS is similar to COVID-19 in etiology and symptoms, thus, the PRRS is considered as the “COVID-19” in the swine world (Zhang et al., 2025). In addition, some ingredients from MXSGS like glycyrrhizin, quercetin, and glycyrrhiza polysaccharides, could exert significant anti-PRRSV effects. Therefore, we suspected that MXSGS can also treat PRRSV (Duan et al., 2015; Yang et al., 2023; Guang et al., 2024).

In our study, we proved that MXSGS administration, whether applied prophylactically or therapeutically, effectively inhibited PRRSV. Furthermore, MXSGS suppressed all stages of the viral life cycle, including attachment, internalization, replication, and release. Mechanistically, MXSGS was found to interact with multiple target proteins through network pharmacology and molecular docking analysis. In short, our research provided a theoretical basis for applying MXSGS in PRRSV infection management.

## 2 Materials and methods

### 2.1 Cells, viruses, reagents

African Green Monkey Kidney Cells (Marc-145 cells) were cultured in Dulbecco’s Modified Eagle’s Medium [DMEM (Gibco, Waltham, MA, USA)], supplemented with 10% fetal bovine serum (FBS); Highly pathogenic type 2 PRRSV (PRRSV-2) strain HuN4-F112 (a Marc-145-adaptive strain) was purchased from Jilin Zhengye Biological Company. Ma-Xing-Shi-Gan-San, a TCM formula composed of Mahuang, Kuxingren, Shigao, and Gancao, was stored in our laboratory and diluted in DMEM.

### 2.2 Cytotoxicity assay

The cytotoxicity of MXSGS was assessed by Cell Counting Kit-8 [CCK-8 (Beyotime, Shanghai, China)]. Marc-145 cells were first seeded into 96-well plates at 1 × 10^5^ cells/mL. Simultaneously, a blank control group was established using only a maintenance medium (DMEM with 2% FBS) without any cells. The MXSGS was serially diluted two-fold in the maintenance medium and then incubated with Marc-145 cells in 96-well plates for 72 h. A negative control group, which consisted solely of the maintenance medium, was also included. After 72 h, 10 μL of CCK-8 reagent was added to each well and incubated at 37°C for 1 h; cytotoxicity was subsequently assessed using the Epoch 2 Microplate Spectrophotometer (BioTek, Winooski, VT, USA).

### 2.3 CPE experiments

Marc-145 cells were inoculated in six-well plates with PRRSV at a multiplicity of infection (MOI) of 0.1 for 1 h, followed by three washes with phosphate-buffered saline (PBS). Subsequently, maintenance medium containing different concentrations of MXSGS (2.25, 4.50, 9.00, and 18.00 mg/mL) were added to the respective wells for co-incubation for 72 h. A negative control group was established without PRRSV inoculation, while a positive control group was infected with PRRSV and supplemented only with maintenance medium. After 72 h, two independent double-blind researchers assessed the cytopathic effect (CPE) using an inverted microscope (Motic, Xiamen, China).

### 2.4 Real-time fluorescence quantitative PCR for RNA extraction

To investigate the inhibitory effect of MXSGS on PRRSV, we extracted total RNA using TRIzol Reagents (Invitrogen, Carlsbad, CA, USA), and the RNA from each sample was subsequently reverse transcribed into cDNA using a reverse transcription kit (ABclone, Woburn, MA, USA). The cDNA was subjected to RT-qPCR analysis employing the SYBR Green Kit (ABclone, Woburn, MA, USA), with *β*-actin selected as the housekeeping gene. The expression levels of PRRSV ORF7 mRNA in different treatment groups were analyzed using the 2^-ΔΔCt^ method. Additionally, the ORF7 mRNA copy number was quantified by absolute quantitative PCR methods (Table 1). The RT-qPCR assay was performed on the qTOWER^3^ real-time PCR system (Analytik Jena, Jena, Germany).

**Table 1:** The sequences of primers used in this study.

Name	Sequence (5′-3′)	The length of the amplified fragment (bp)
ORF7-F	GGAGAAGCCCCATTTCCCTC	130
ORF7-R	TGACAGGGCACAAGTTCCAG	
β-actin-F	CTATGTCGCCCTGGACTTCG	157
β-actin-R	CATGCCCAGGAAGGAAGGTT	

F, forward primer; R, reverse primer.

### 2.5 Viral titer calculation

Marc-145 cells seeded in 96-well plates were infected with serial 10-fold dilutions of samples in eight replicates, incubated at 37°C with 5% CO2 for 5 days; meanwhile, a negative control group was established without viral exposure. After incubation, the cytopathic effects in each well were assessed to calculate PRRSV viral titer based on the Reed-Muench method (Pizzi, 1950). The results were expressed as 50% tissue culture infective dose (TCID_50_)/mL.

### 2.6 Anti-viral assay

#### 2.6.1 Full course of administration

Marc-145 cells seeded in six-well plates were incubated with a maintenance medium containing low (4.5 mg/mL), medium (9 mg/mL), and high (18 mg/mL) concentrations of MXSGS for 8 h and washed three times with PBS. The maintenance medium containing different MXSGS concentrations was mixed with PRRSV at a multiplicity of infection (MOI) of 0.2 in equal volumes and co-incubated for 1 h at 37°C. Subsequently, the mixture was inoculated onto Marc-145 cells for 1 h and washed three times with PBS. Next, the maintenance medium containing varying concentrations of MXSGS was added to the Marc-145 cells and incubated for 48 and 72 h. A control group consisting solely of maintenance medium without MXSGS was also established. Finally, the relative expression levels of PRRSV mRNA and viral titer in the different treatment groups were assessed using RT-qPCR and TCID_50_ assays.

#### 2.6.2 Prophylactic administration (drug administration before PRRSV inoculation)

Marc-145 cells seeded in six-well plates were incubated with a maintenance medium containing low, medium, and high concentrations of MXSGS for 8 h; concurrently, a control group was incubated solely with a maintenance medium. Subsequently, the cells were washed three times with PBS, inoculated with PRRSV (MOI = 0.1), and then incubated for 1 h before replacing the medium to continue incubating for 72 h. The relative expression levels of PRRSV mRNA and viral titer in different groups were assessed using RT-qPCR and TCID_50_ assays.

#### 2.6.3 Direct virucidal administration (simultaneous drug administration and PRRSV inoculation)

The maintenance medium containing low, medium, and high concentrations of MXSGS was mixed with PRRSV (MOI = 0.2) in equal volumes and co-incubated at 37°C for 1 h, followed by inoculation onto Marc-145 cells for 1 h. Meanwhile, a control group of virus incubated with a maintenance medium was established. After incubation, the cells were washed three times with PBS and replaced with a maintenance medium to continue incubation for 72 h. The relative expression levels of PRRSV mRNA and viral titer in different groups were detected using RT-qPCR and TCID_50_ assays.

#### 2.6.4 Therapeutic administration (drug administration post PRRSV inoculation)

Marc-145 cells were infected with PRRSV (MOI = 0.1) for 1 h, followed by three washes with PBS, and then incubated in a maintenance medium containing low, medium, and high concentrations of MXSGS for 72 h. Meanwhile, a control group was established and incubated solely with a maintenance medium. The relative expression levels of PRRSV mRNA and viral titer in different groups were assessed using RT-qPCR and TCID_50_ assays.

#### 2.6.5 Impact on virus attachment

Marc-145 cells seeded in six-well plates were pre-cooled at 4°C for 1 h after which maintenance medium containing MXSGS was added, followed by immediate inoculation with PRRSV (MOI = 1) and attachment at 4°C for 1 h (during which the virus could attach but not enter the cells). The cells were washed three times with pre-cooled PBS. Meanwhile, a control group was established and incubated solely with a maintenance medium. Finally, the cells were lysed for RNA extraction and relative quantitative RT-qPCR analysis.

#### 2.6.6 Impact on virus internalization

Marc-145 cells were pre-cooled at 4°C for 1 h. The cells were washed three times with pre-cooled PBS, inoculated with PRRSV (MOI = 1) at 4°C for 1 h, and subsequently washed three times with PBS. Following this, a maintenance medium containing MXSGS was added, while a control group consisting solely of maintenance medium was established. Finally, the cells were incubated at 37°C for 3 h before being lysed for RNA extraction and RT-qPCR analysis.

#### 2.6.7 Impact on virus replication

Marc-145 cells were inoculated with PRRSV (MOI = 0.1) at 37°C for 1 h, followed by three washes with PBS, and then incubated in a maintenance medium for 6 h. Subsequently, a maintenance medium containing MXSGS was added to the cells, while a control group consisting solely of maintenance medium was established. Afterward, the cells were lysed for RNA extraction and RT-qPCR analysis at 7 h post-infection (h. p. i.), 8 h. p. i., and 9 h. p. i., respectively. The expression levels from each treatment group were normalized and compared to those of the control group at 7 h. p. i.

#### 2.6.8 Impact on virus release

Marc-145 cells were inoculated with PRRSV (MOI = 0.1) at 37°C for 1 h, followed by three washes with PBS, and then incubated in a maintenance medium for 18 h. Subsequently, a maintenance medium containing MXSGS was added, while a control group consisting solely of maintenance medium was established. Finally, 20 min, 40 min, and 60 min after drug administration, RNA was extracted from the cell supernatants, and the copy number of PRRSV ORF7 mRNA was quantified using absolute quantitative PCR methods.

### 2.7 Screening active ingredients of MXSGS

The TCMSP database^1^ (Ru et al., 2014) was utilized to extract the constituents of the Mahuang, Kuxingren, and Gancao. The criteria for screening in TCMSP were set as oral bioavailability (OB) > 30% and drug-likeness (DL) > 0.18 (Chang et al., 2024). Each constituent’s simplified molecular linear input specification (SMILES) numbers were collected through the PubChem database.^2^ These SMILES were employed to screen for active ingredients on the SwissADME platform,^3^ with a gastrointestinal absorption score classified as “high” and a drug-likeness rating of at least 3 “Yes” responses. Subsequently, the SwissTargetPrediction platform^4^ was used to predict the targets corresponding to the active ingredient of Mahuang, Kuxingren, and Gancao. Since Shigao is not included in the TCMSP database, calcium (Ca), magnesium (Mg), iron (Fe), potassium (K), and sodium (Na) were selected as primary components based on previous literature (Liu et al., 2024), and their targets were collected in the STITCH 5.0 database.^5^ Finally, targets associated with Mahuang, Kuxingren, Gancao, and Shigao were combined while qualifying species as “*Sus scrofa*” for screening; target names were standardized using the UniProt database.^6^

### 2.8 Screening PRRSV disease targets

To identify the PRRSV disease targets, the keyword “porcine reproductive and respiratory syndrome” was queried in the GeneCards,^7^ OMIM^8^ and PharmGKB database.^9^ These databases illuminate the relationship between targets and diseases from various perspectives. Then, disease targets were consolidated, and the species was qualified as “*sus scrofa*” to filter the validated targets and standardize the target nomenclature through the UniProt database (accessed on 15 September 2024).

### 2.9 Construction of protein–protein interaction network and “MXSGS ingredient-PRRSV-targets network”

The targets of the active ingredients in MXSGS were compared with the disease targets associated with PRRSV to identify common targets, which were defined as direct therapeutic targets acting on PRRSV with MXSGS. The common targets were utilized to construct a target network via the String database^10^ (Doncheva et al., 2019), with species restricted to “*Sus scrofa*.” Subsequently, a protein–protein interaction (PPI) network was generated using Cytoscape version 3.7.1 (Shannon et al., 2003). Topological analysis of the intersected targets was conducted based on UnDir degree, betweenness centrality, and closeness centrality to screen core potential targets for MXSGS treatment of PRRSV.

Subsequently, the active ingredients and common potential targets were imported into Cytoscape version 3.7.1 to construct the “MXSGS ingredient-PRRSV-targets network,” filtering for key active ingredients of MXSGS based on UnDir degree by Analyze Network plug-in.

### 2.10 GO and KEGG analysis

The core targets of MXSGS against PRRSV, identified from the 2.9 analysis, were input into the DAVID database^11^ (Dennis et al., 2003), with the species set to “*Sus scrofa*.” Gene Ontology (GO) and Kyoto Encyclopedia of Genes and Genomes (KEGG) pathway analyses were performed. The top 20 GO enrichment results and KEGG pathways were selected for visualization using an online bioinformatics tool.^12^ Then, the KEGG analysis results were imported into Cytoscape version 3.7.1 for topological analysis to construct the “MXSGS-PRRSV-targets-pathways network.”

### 2.11 Molecular docking

The top 6 potential core targets were identified from the PPI network and the “MXSGS-PRRSV-targets-pathways network, “respectively. The active ingredients with the highest 5 UnDir degrees were selected from the “MXSGS ingredient-PRRSV-targets network.” All screened targets and ingredients were utilized for molecular docking validation. The 3D structures of ALB (PDB ID: 4LA0), PPARG (PDB ID: 9CK0), CASP3 (PDB ID: 1RE1), STAT3 (PDB ID: 6NJS), TGFB1 (PDB ID: 6UJA), JAK2 (PDB ID: 7F7W), TLR4 (PDB ID: 2Z66), PRKACA (PDB ID: 7Y1G), and PRKACB (Prediction with AlphaFold) were downloaded from the PDB database.^13^ Subsequently, water molecules and small molecule ligands were removed from core target proteins by PyMOL version 2.6.0. Charge balancing and hydrogenation were performed with AutoDock Tools version 1.5.7 software, after which the processed protein targets were converted into .pdbqt format files.

Meanwhile, the .mol2 structure of the active ingredient was downloaded through the TCSMP database (accessed on 30 September 2024) and converted into the .pdbqt format files using AutoDock Tools 1.5.7 software. Next, molecular docking was conducted with AutoDock Vina software to obtain binding energy affinity. Specifically, affinity < −4.25 kcal/mol indicates the presence of binding activity between ligand and target; affinity < −5.0  kcal/mol implies good binding activity; affinity < −7.0 kcal/mol suggests strong docking activity (Ji et al., 2023).

Finally, the optimal conformations of the key active ingredient in MXSGS and the core target protein were visualized using PyMOL version 2.6.0.

### 2.12 Statistical analysis

GraphPad Prism 10.1.2 software (GraphPad Software, La Jolla, CA, USA) was used for data analysis. Differences between groups were assessed using the two-tailed Student’s *t*-test, one-way ANOVA, or two-way ANOVA analysis.

## 3 Results

### 3.1 MXSGS exhibits anti-PRRSV activity

**Figure 1:**
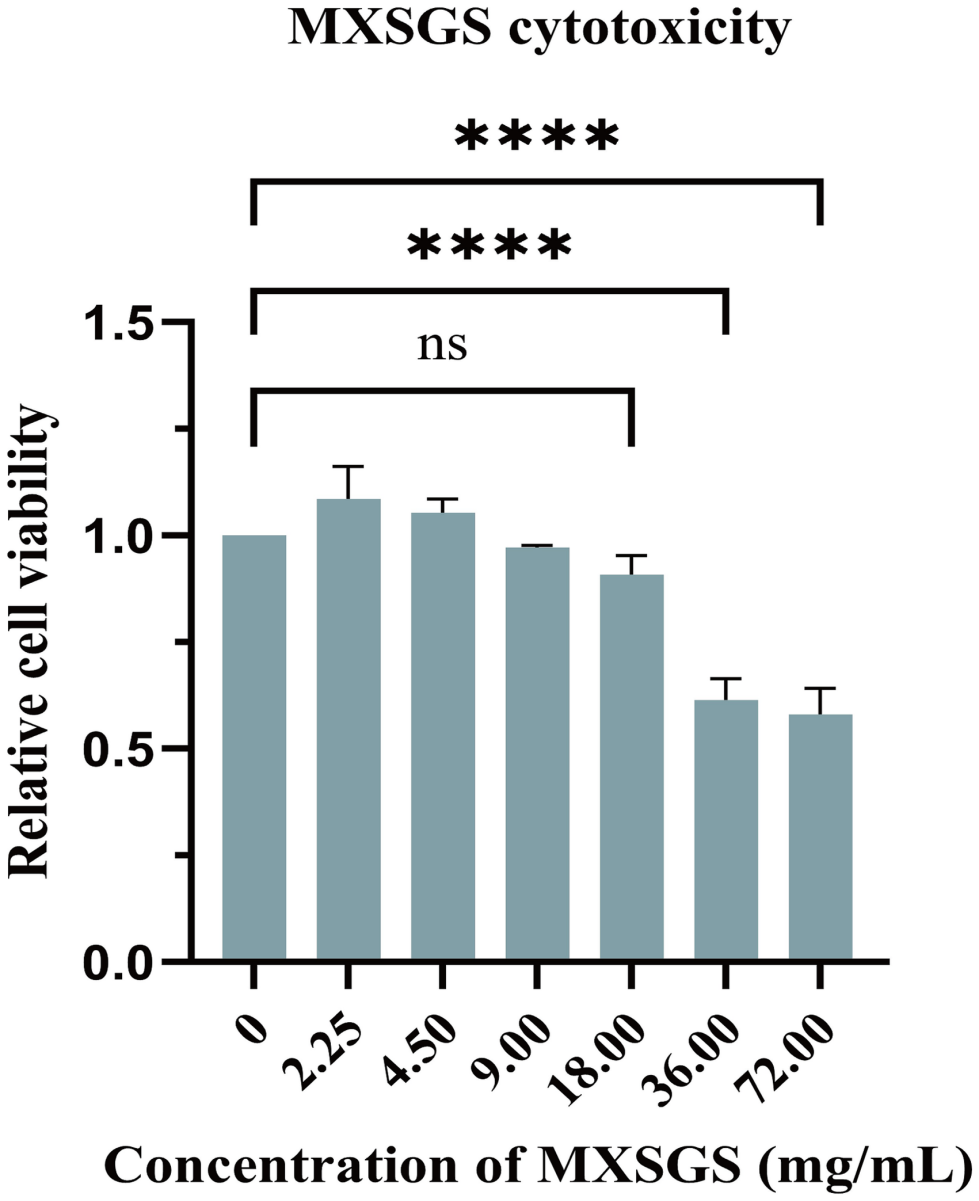
Cytotoxicity of Ma-Xing-Shi-Gan-San (MXSGS) on Marc-145 cells. Different concentrations (0.56, 1.13, 2.25, 4.50, 9.00, 18.00, 36.00, 72.00 mg/mL) of MXSGS were incubated with Marc-145 cells for 72 h, followed by the addition of CCK-8 reagent to assess cell viability through measurement of OD450 absorbance across various groups. A negative control group was established using a maintenance medium without MXSGS (0 mg/mL). Data were performed as the means and standard deviations from three independent experiments and analyzed using one-way ANOVA. ns, no significance; ****, *p* < 0.0001.

Firstly, we determined the safety concentration of MXSGS on Marc-145 cells using the CCK-8 assay (Figure 1). The results indicated that MXSGS did not significantly reduce cell viability at concentrations below 18.00 mg/mL.

**Figure 2:**
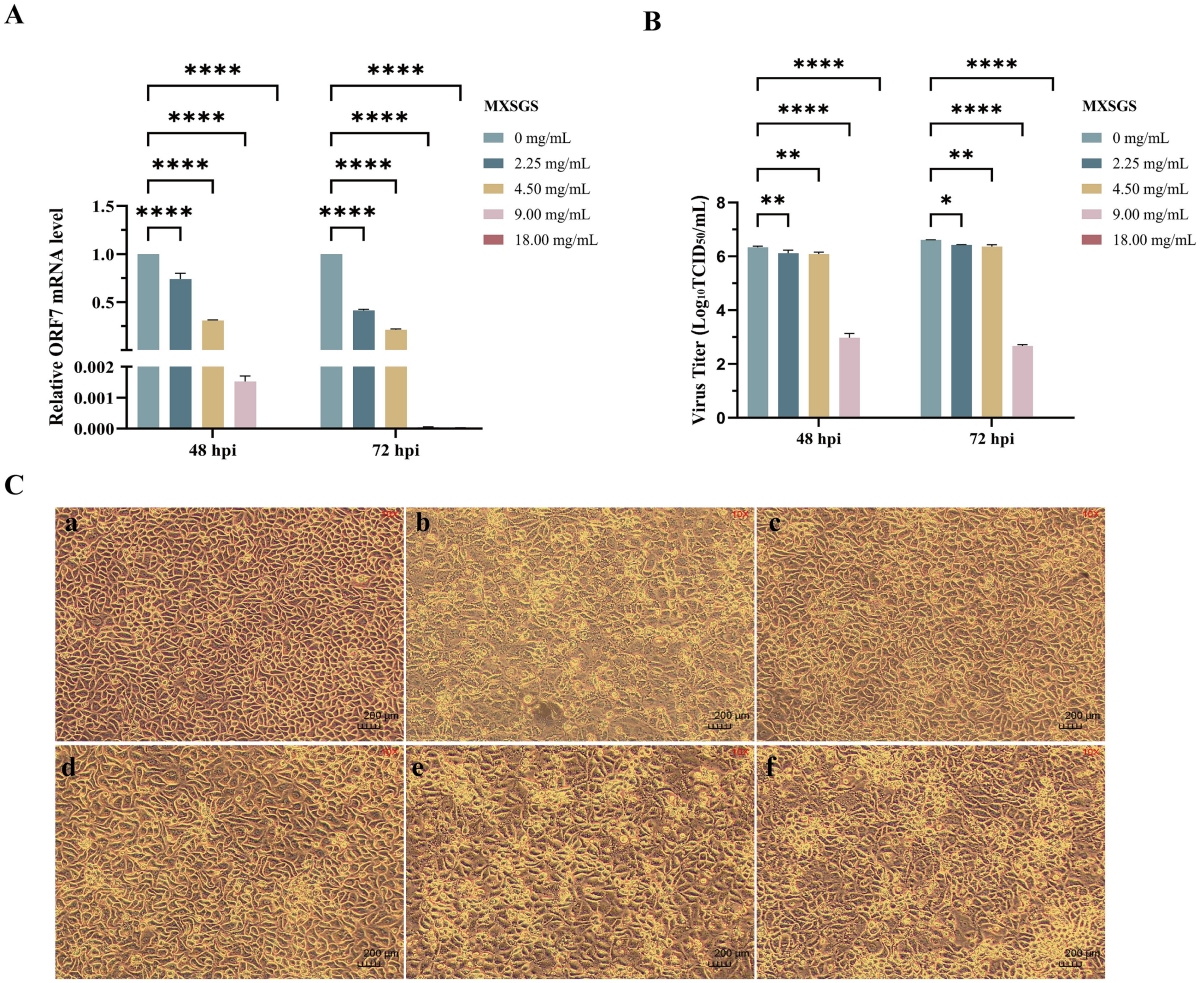
MXSGS could inhibit the porcine reproductive and respiratory syndrome virus (PRRSV) *in vitro*. The extent of cytopathic effect (CPE), the relative expression levels of PRRSV ORF7 mRNA, and the viral titers were assessed and compared with a positive control group (PRRSV + 0 mg/mL MXSGS), as detailed in Methods 2.3 and 2.6.1. **(a)** Relative PRRSV ORF7 mRNA expression levels in each group compared to the control group; **(b)** PRRSV titration using the TCID_50_ assay; **(c)** MXSGS inhibited PRRSV-induced CPE. Image panels: **(a)** Cell control group; **(b)** Virus group; **(c)** PRRSV + 18.00 mg/mL; **(d)** PRRSV + 9.00 mg/mL; **(e)** PRRSV + 4.50 mg/mL MXSGS; **(f)** PRRSV + 2.25 mg/mL. Data were performed as the means and standard deviations from three independent experiments and analyzed using one-way ANOVA. *, 0.01 ≤ *p* < 0.05; **, 0.001 ≤ *p* < 0.01; ****, *p* < 0.0001.

Then, MXSGS demonstrated a significant dose-dependent reduction in PRRSV ORF7 mRNA levels in Marc-145 cells. For instance, there was a 655-fold and a 2 × 10^4^-fold decrease in PRRSV mRNA levels with 9.00 mg/mL MXSGS from 48 h. p. i. to 72 h. p. i., respectively (Figure 2A). Similarly, MXSGS significantly reduced PRRSV titers in a concentration-dependent manner; specifically, the group treated with 9.00 mg/mL MXSGS exhibited reductions of 10^3.36^-fold and 10^3.94^-fold in PRRSV titers after incubation for 48 h and 72 h, respectively, compared to the control group (Figure 2B).

Additionally, we investigated whether MXSGS could mitigate the cytopathic effect (CPE) induced by PRRSV in Marc-145 cells. The CPE in the virus group was evident, characterized by rounded, detached cells that aggregated into clumps. In contrast, the CPE was significantly alleviated in the MXSGS treatment groups (2.25, 4.50, 9.00, and 18.00 mg/mL), demonstrating a dose-dependent relationship (Figure 2C). These results indicate that MXSGS can effectively inhibit PRRSV infection in Marc-145 cells.

### 3.2 MXSGS inhibits PRRSV through therapeutic and prophylactic administration

**Figure 3:**
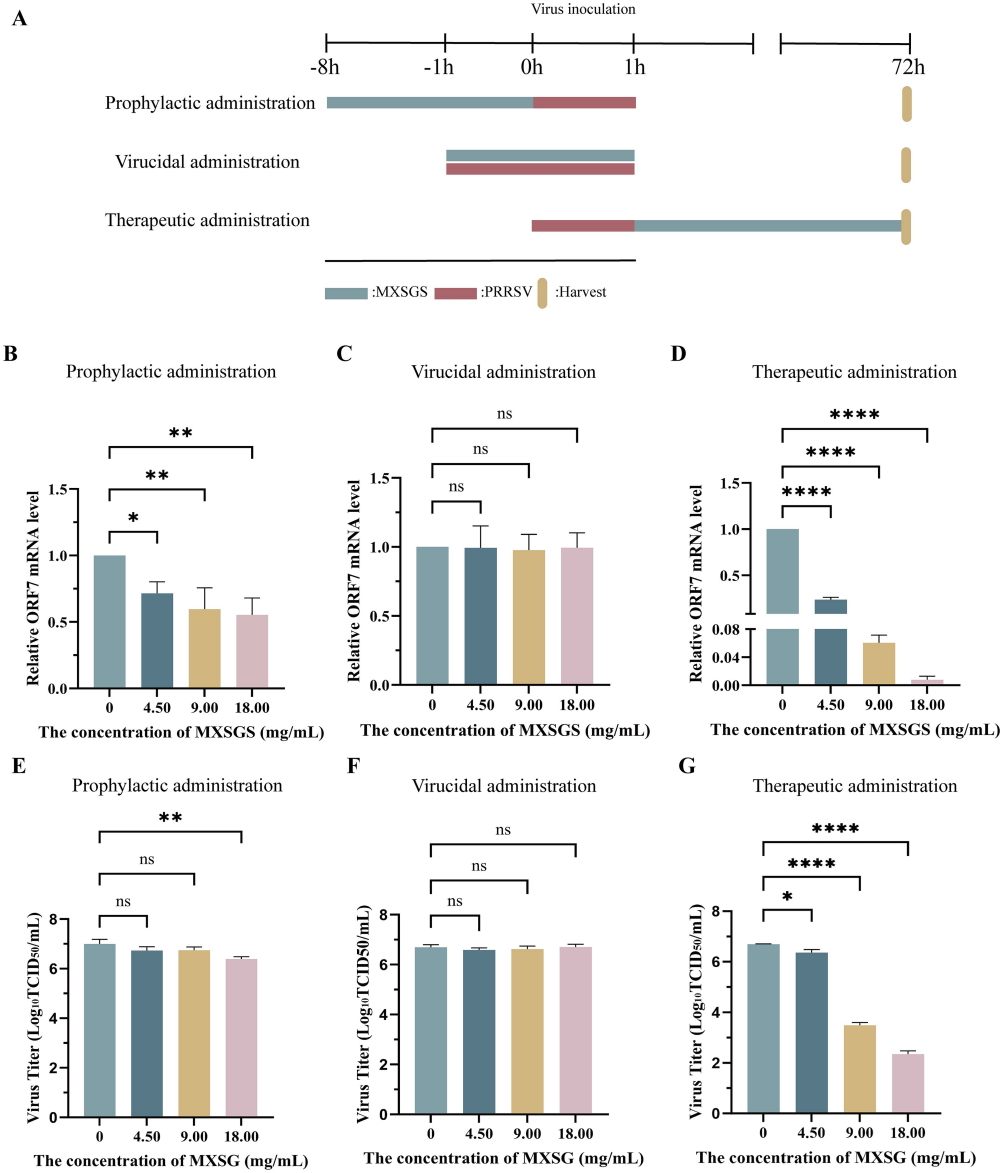
MXSGS exhibited anti-PRRSV activities through prophylactic and therapeutic administration. The experiment followed the protocols outlined in Methods 2.6.2, 2.6.3, and 2.6.4, with a control group (MXSGS at 0 mg/mL) consisting solely of maintenance medium. The PRRSV mRNA expression levels and viral titers for each group were assessed using RT-qPCR and TCID_50_ assays. **(A)** The schematic diagram illustrates the various stages of MXSGS administration during PRRSV infection; **(B–D)** relative PRRSV mRNA expression levels in **(B)** prophylactic administration. **(C)** direct virucidal administration, and **(D)** therapeutic administration, respectively; **(E-G)** changes in PRRSV viral titers corresponding to **(E)** prophylactic administration, **(F)** direct virucidal administration, and **(G)** therapeutic administration, respectively. Data were performed as the means and standard deviations from three independent experiments and analyzed using one-way ANOVA. ns, no significance; *, 0.01 ≤ *p* < 0.05; **, 0.001 ≤ *p* < 0.01; ****, *p* < 0.0001.

To further investigate the impact of MXSGS administration on PRRSV, three groups with different treatment stages were established (Figure 3A). Firstly, the PRRSV ORF7 mRNA level was reduced by nearly half in the prophylactic administration group (Figure 3B). The viral titer was significantly decreased only in the high-dose MXSGS group (18.00 mg/mL), while no significant reduction in PRRSV titers was observed in the middle and low-dose groups (9.00 and 4.50 mg/mL) (Figure 3E). Secondly, all doses of MXSGS administered in the direct virucidal group exhibited no significant inhibition of PRRSV mRNA expression levels and viral titers (Figures 3C,F). Finally, the therapeutic administration group demonstrated reductions of 3.26-fold, 15.57-fold, and 126.84-fold in PRRSV mRNA expression levels (Figure 3D), along with cuts of 1.12-fold, 10^3.2^-fold, and 10^4.34^-fold in viral titers (Figure 3G) for low-dose, medium-dose, and high-dose groups of MXSGS, respectively. Hence, both prophylactic and therapeutic administration modes inhibited PRRSV proliferation. Notably, the therapeutic administration group exhibited the most pronounced anti-PRRSV effects.

### 3.3 MXSGS suppressed all phases of the PRRSV replication cycle

**Figure 4:**
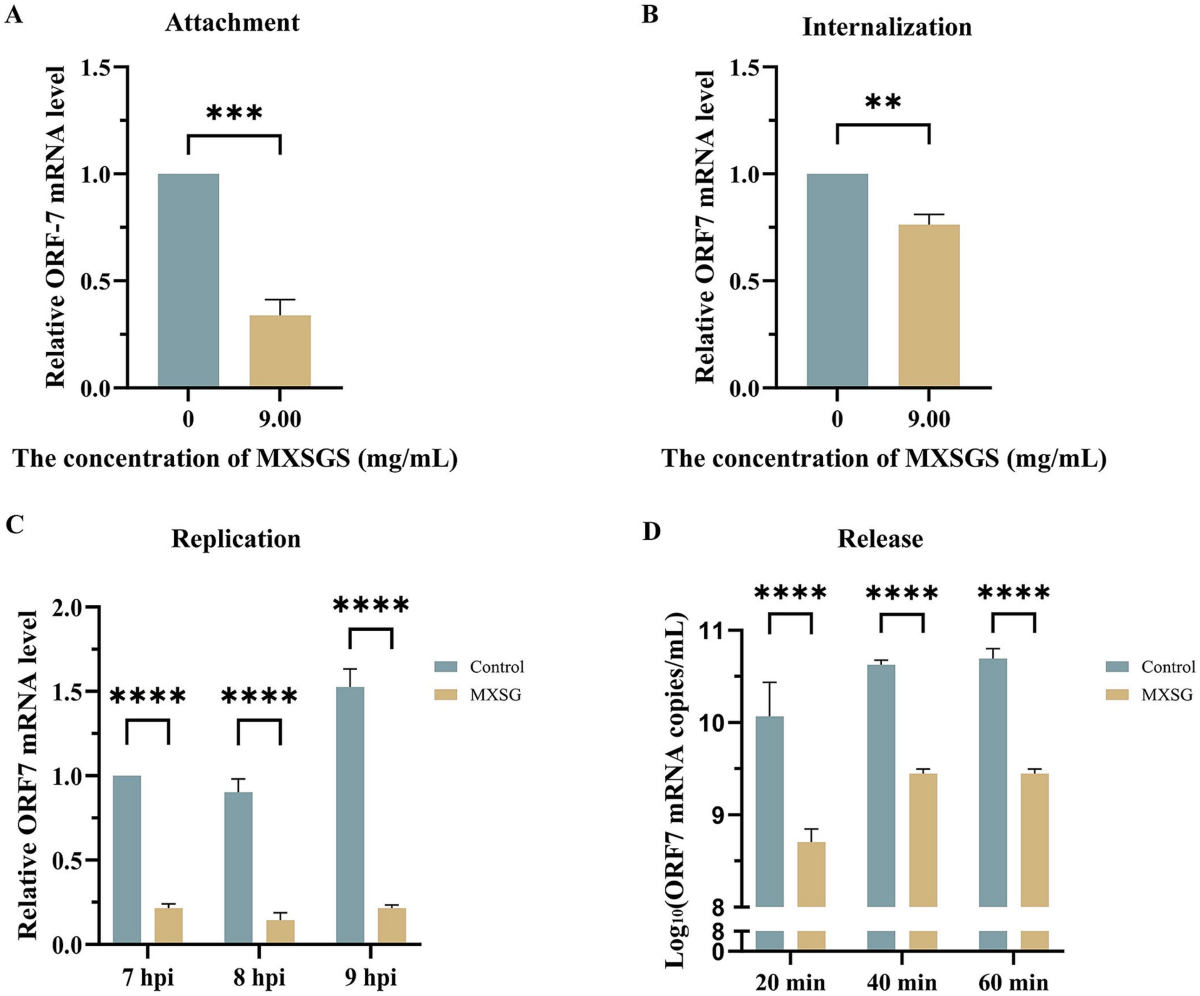
MXSGS significantly restricted PRRSV by targeting the whole viral life cycle. Marc-145 cells were incubated with MXSGS (9.00 mg/mL) and PRRSV as described in Method 2.6.5 **(A)**, Method 2.6.6 **(B)**, Method 2.6.7 **(C)**, and Method 2.6.8 **(D)** of the Section 2. Data were performed as the means and standard deviations from three independent experiments and analyzed using two-tailed Student’s *t*-test **(A,B)** or two-way ANOVA **(C,D)**. **, 0.001 ≤ *p* < 0.01; ***, 0.0001 ≤ *p* < 0.001; ****, *p* < 0.0001.

To investigate the mechanism of MXSGS against PRRSV, we examined its effects on each phase of the viral life cycle: attachment, internalization, replication, and release. Firstly, pre-cooled Marc-145 cells were inoculated with PRRSV (MOI = 1) after adding MXSGS (9.00 mg/mL) and incubated at 4°C for 1 h. The results from RT-qPCR indicated that PRRSV attachment was reduced by 0.31-fold in the MXSGS group (Figure 4A). Secondly, pre-cooled cells were infected with PRRSV for 1 h at 4°C before being treated with MXSGS (9.00 mg/mL) for an additional 3 h at 37°C; this treatment resulted in a 1.95-fold reduction in the amount of PRRSV internalized into Marc-145 cells (Figure 4B). Thirdly, PRRSV-infected Marc-145 cells were switched to incubation with MXSGS (9.00 mg/mL) at 6 h. p. i., leading to reductions in PRRSV ORF7 mRNA levels by factors of 3.65-fold, 5.26-fold, and 6.10-fold at 7, 8, and 9 h. p. i., respectively (Figure 4C). Finally, after switching to incubation with MXSGS (9.00 mg/mL) at 18 h. p. i., the copy numbers of PRRSV ORF7 mRNA in cell supernatants decreased by 26.10-fold, 17.27-fold, and 16.73-fold after drug intervals of 20 min, 40 min, and 60 min, respectively. In summary, MXSGS inhibited all phases of the PRRSV life cycle, including attachment, internalization, replication, and release.

### 3.4 Network pharmacological analysis of potential anti-PRRSV targets of MXSGS

**Figure 5:**
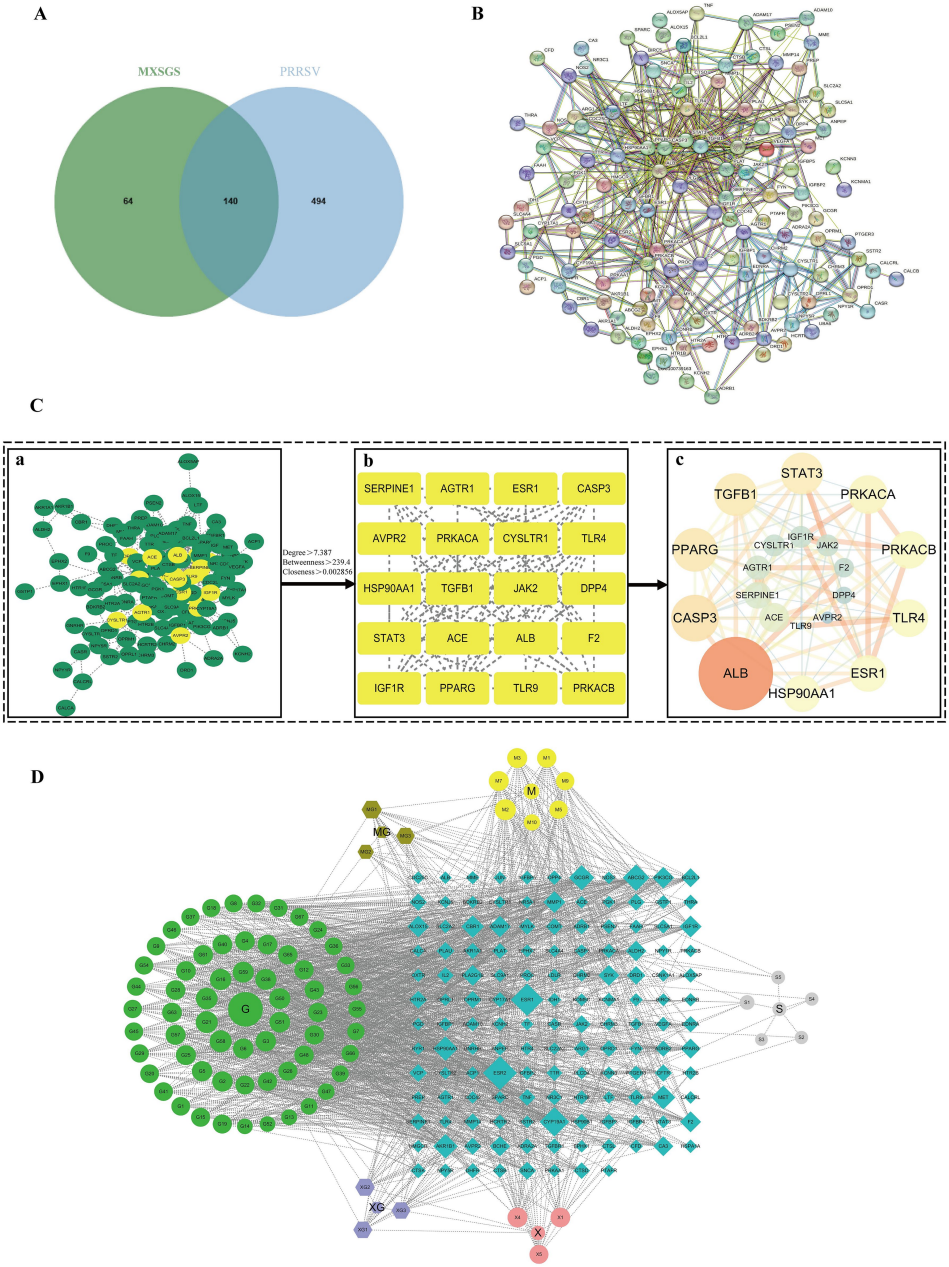
Network pharmacological analysis of potential target networks of MXSGS against PRRSV. **(A)** Venn diagram illustrating the intersection of common targets between MXSGS (as a therapeutic agent) and PRRSV (as a disease). **(B)** PPI network of common targets generated by the STRING database **(C)** The process of obtaining core targets of MXSGS anti-PRRSV from PPI networks: **(a)** network diagram obtained by Cytoscape version 3.7.1; **(b)** the screened core targets after topological analysis; **(c)** the rearranged network diagram based on unDir degree and binding score, respectively. Node size is represented in ascending order according to unDir degree, while node color reflects ascending order based on union score. **(D)** The network diagram represents the MXSGS ingredients and their corresponding anti-PRRSV targets. The green circles, yellow circles, grey circles, and orange-red circles represent components of Gancao, Mahuang, Shigao, and Kuxingren, respectively. The light-purple octagonal shapes depict shared ingredients between Kuxingren and Gancao, brown octagonal shapes illustrate shared ingredients between Mahuang and Gancao, and blue diamonds represent intersecting targets among common targets of both MXSGS and PRRSV.

We initially screened 82 ingredients (Supplementary Table S1) from MXSGS, identified 204 UniProt-validated targets associated with MXSGS (Supplementary Table S2), and obtained 634 disease-related targets after merging and removing duplicates, which included 606, 111, and 8 PRRSV targets sourced from the GeneCards, PharmGKB, and OMIM databases, respectively (Supplementary Table S3). Then, we selected 140 targets at the intersection of MXSGS and PRRSV targets (Supplementary Table S4; Figure 5A), constructing a Protein–Protein Interaction (PPI) network using the STRING database (Figure 5B) and Cytoscape version 3.7.1. The PPI network was then subjected to topological analysis via the Centiscape version 2.2 plug-in, which identified 20 core targets; among these, the top 6 included ALB (UnDir value = 56), PPARG (UnDir value = 30), CASP3 (UnDir value = 27), STAT3 (UnDir value = 26), TGFB1 (UnDir value = 25), and PRKACA (UnDir value = 22) (Figure 5C). Additionally, we constructed the “MXSGS ingredient-PRRSV-targets network” (Figure 5D), which illustrates that MXSGS comprises multiple components, with each ingredient corresponding to various targets. Subsequently, the top 5 active small molecules within the MXSGS ingredients were identified based on their UnDir degree: Calycosin (UnDir value = 29), Odoratin (UnDir value = 29), Glyzaglabrin (UnDir value = 28), 7,2′,4′-trihydroxy-5-methoxy-3-arylcoumarin (UnDir value = 27), and Eriodictyol (UnDir value = 26).

**Figure 6:**
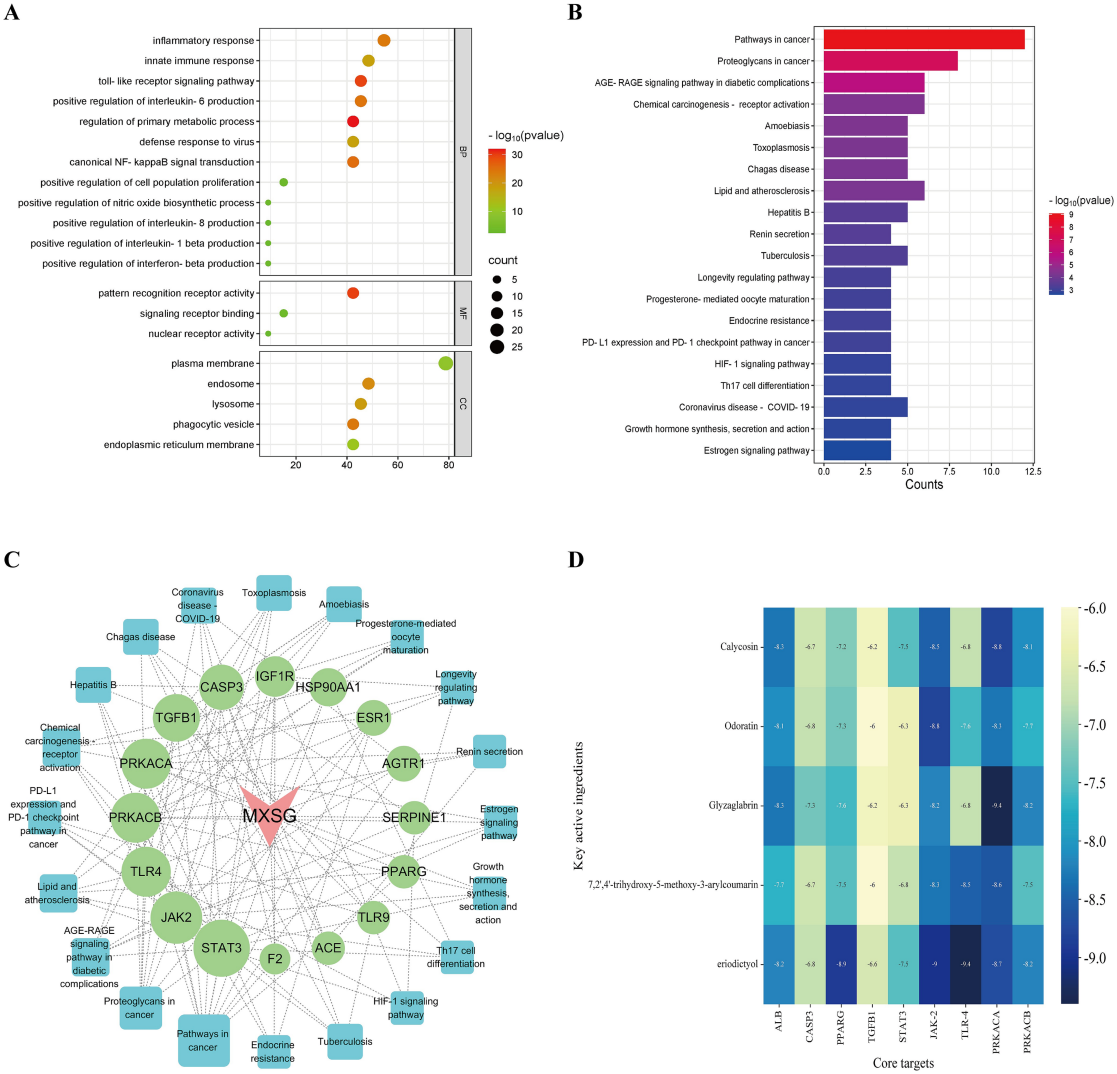
GO and KEGG analyses, along with molecular docking studies. **(A)** Visualization of the top 20 GO enrichment results for core targets. **(B)** Visualization of the top 20 KEGG enrichment results for core targets. **(C)** MXSGS-PRRSV-targets-pathways network: red arrows indicate MXSGS, green circles represent core targets derived from the PPI network, and blue squares denote pathways identified through KEGG enrichment analysis. **(D)** The binding energy thermogram heat map illustrates the binding energies from molecular docking between the top 5 key active ingredients of MXSGS (arranged vertically) and a total of 9 core targets (placed horizontally) obtained from the networks above.

Subsequently, Gene Ontology (GO) and Kyoto Encyclopedia of Genes and Genomes (KEGG) pathway analyses were conducted on the 20 core targets identified from the PPI network analysis. The GO analysis yielded a total of 52 entries (p<0.05), comprising 38 related to biological processes (BP), 5 of molecular functions (MF), and 9 associated with cellular components (CC). The top 20 GO enrichment results were visualized (Figure 6A), indicating that MXSGS could inhibit PRRSV through Toll-like receptor signaling pathway, typical NF-κB signaling, positive regulation of interleukin-6 production, inflammatory response, viral defense response, and intrinsic immune response, etc. In addition, KEGG enrichment analysis revealed a total of 61 pathways (*p <* 0.05). The results of the top 20 KEGG pathways (Figure 6B) showed that the effection of MXSGS in anti-PRRSV primarily enriched in pathways in cancer, the AGE-RAGE signaling pathway, associated with diabetic complications, Th17 cell differentiation, coronavirus disease-pneumococcal pneumonia, etc.

Utilizing these selected KEGG pathways, we constructed the “MXSGS-PRRSV-targets-pathways network” (Figure 6C). Through topological analysis, 6 key targets including STAT3 (UnDir value = 14), JAK2 (UnDir value = 12), TLR4 (UnDir value = 11), PRKACB (UnDir value = 11), PRKACA (UnDir value = 11), and TGFB1 (UnDir value = 10) were identified. In summary, a total of 9 core targets of MXSGS against PRRSV, ALB, PPARG, CASP3, STAT3, TGFB1, JAK2, TLR4, PRKACA, and PRKACB were obtained from the above two networks for molecular docking analysis.

### 3.5 Molecular docking validation of potential targets

**Figure 7:**
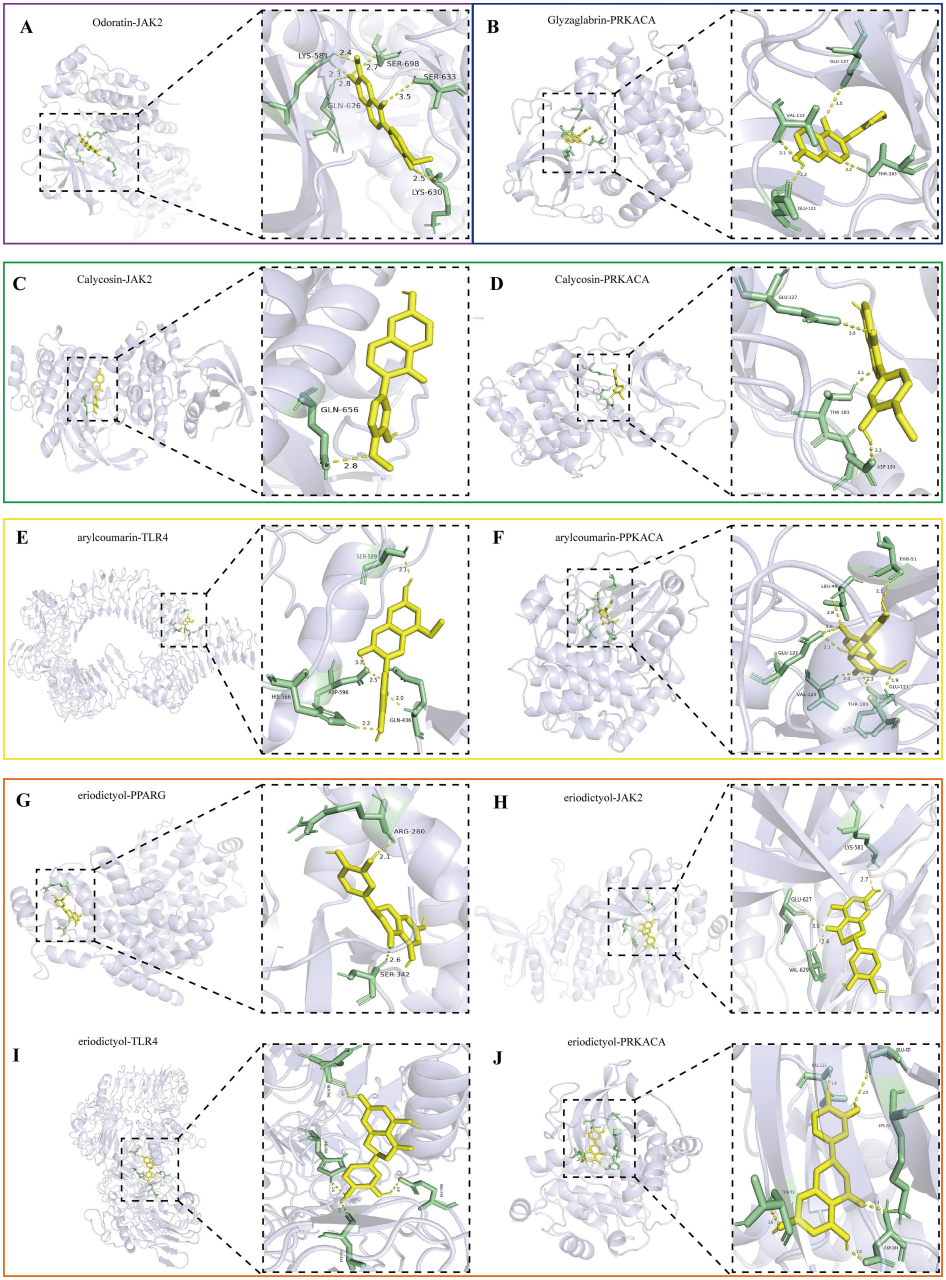
Visualization of the top 10 binding energy of docking between the key active ingredient of MXSGS and core target proteins of PRRSV by Pymol version 2.6.0, including **(A)** Odoratin and JAK2. **(B)** Glyzaglabrin and PRKACA. **(C)** Calycosina and JAK2. **(D)** Calycosin and PRKACA. **(E)** 7,2′,4′-trihydroxy-5-methoxy-3-arylcoumarin andTLR4. **(F)** 7,2′,4′-trihydroxy-5-methoxy-3-arylcoumarin and PRKACA. **(G)** Eriodictyol and PPARG. **(H)** Eriodictyol and JAK2. **(I)** Eriodictyol and TLR4. **(J)** Eriodictyol and PRKACA.

The top 5 active ingredients of MXSGS (acting as ligands) and a total of 9 core targets (serving as receptors) were utilized for molecular docking analysis, where all binding energies were lower than −6.0 kcal/mol, indicating an excellent molecular docking effect (Figure 6D). Lower binding energy values suggest more stable molecular ligand and protein receptor conformations. Among these, 10 ligand-receptor pairs exhibited high binding affinity with binding energies lower than −8.5 kcal/mol; we visualized these optimal conformations using PyMOL version 2.6.0 (Figure 7).

Interaction forces were observed with at least 1 hydrogen bond in all 10 ligand-receptor pairs. For instance, Odoratin exhibited a favorable interaction with the JAK2 protein, characterized by an absolute binding energy value of −8.8 kcal/mol and the formation of 6 hydrogen bonds at the docking site, with an average bond length of 2.7 Å. The connection position situated at the JAK2 protein base acid residues, namely LYS-581A, GLN-626A, LYS-630A, SER-633A, and SER-698A (Figure 7A). The binding energy between Glyzaglabrin and PRKACA was −9.4 kcal/mol, forming 4 hydrogen bonds with an average bond length of 2.31 Å. The connection position was identified at the amino acid residues GLU-121A, VAL-123A, GLU-127A, and THR-183A (Figure 7B). Additionally, 7,2′,4′-trihydroxy-5-methoxy-3-arylcoumarin interacting with PRKACA noted binding energy of −8.6 kcal/mol while generating 7 hydrogen bonds at the docking position with an average bond length of 2.5 Å; this connection involved amino acid residues LEU-49A, THR-51A, GLU-121A, VAL-123A, GLU-127A, and THR-183A (Figure 7F).

## 4 Discussion

PRRS and COVID-19 are viral lung infections sharing symptoms like lung inflammation, respiratory distress, and fatal outcomes. Notably, MXSGS has shown efficacy in treating lung diseases like COVID-19 and H1N1 (Wang et al., 2011; Huang et al., 2021), In our study, the broad-spectrum antiviral properties of MXSGS extended effectiveness to PRRS as well. After demonstrating the anti-PRRSV ability of MXSGS *in vitro*, we showed that MXSGS exerts direct antiviral effects via targeting all stages of the PRRSV life cycle, including attachment, internalization, replication, and release. Many TCMs also exert antiviral effects on PRRSV through indirect mechanisms such as immune regulation or inhibition of pathogenic pathways via cytokine modulation (Bello-Onaghise et al., 2020). For instance, quercetin ameliorated PRRSV-induced inflammation by inhibiting TGF-*α*, IL-1*β*, and IL-6 levels by regulating arachidonic acid and glutamine metabolism (Guang et al., 2024). Dipotassium glycyrrhizinate was shown to promote the production of IFN-α, IFN-β, and IL-1β in PAM cells while inhibiting PRRSV N protein expression; this led to an inhibition of viral particle assembly (Wang et al., 2013). Therefore, we suspected that MXSGS may also exert anti-PRRSV properties via a similar indirect anti-viral role in prophylactic and therapeutic administration, which requires further trials to verify.

To compensate for the limitation of experiments, we further applied network pharmacology and molecular docking techniques to analyze the molecular mechanism of MXSGS inhibition of PRRSV. Network pharmacology represents a novel approach to elucidating the effects and mechanisms of therapeutic agents, offering a comprehensive methodological perspective for the holistic screening of traditional medicine and target identification (Yu et al., 2024). Molecular docking, which involves the design of agents through receptor characterization and analysis of interactions between receptors and agent molecules, has emerged as a pivotal technique in computer-aided drug research in recent years (Paggi et al., 2024). Utilizing these analytical methods, 82 active ingredients of MXSGS and 140 potential anti-PRRSV targets were screened. Most targets were connected to two or more herbs by mapping the “MXSGS ingredient-PRRSV-targets network,” and there were numerous overlapping targets across multiple ingredients that might produce synergistic effects.

The top 5 active ingredients of MXSGS were selected, including Calycosin, Odoratin, Glyzaglabrin, 7,2′,4′-trihydroxy-5-methoxy-3-arylcoumarin, and Eriodictyol. Specifically, calycosin, a member of the class of 7-hydroxy isoflavones, is known for its anti-inflammatory and anti-cancer properties, and it was also found to have significant antiviral activity against coxsackie virus B₃ (CVB₃) and human immunodeficiency virus (HIV) in vitro (Chen et al., 2011; Deng et al., 2021; Zhang et al., 2024). Odoratin belongs to the flavone and has demonstrated a significant transactivation effect on a PPARG to play a crucial role in anti-inflammatory and antiviral activity (Zhang et al., 2012). Eriodictyol, a polyphenolic flavanone exhibiting anti-inflammatory, antipyretic, and antioxidant activity, was shown to have strong coronavirus inhibitory properties by inhibiting inflammatory mediator release from neuro-COVID-associated mast cells and activated microglia (Deng et al., 2020; Theoharides and Kempuraj, 2023; Yin et al., 2024). In addition, Eriodictyol could bind to almost all selected core targets with good binding energy, suggesting its importance in treating PRRSV. Therefore, these active ingredients within MXSGS might inhibit PRRSV proliferation by suppressing similar inflammatory processes.

Subsequently, through the analysis of the PPI network and the “MXSGS ingredient-PRRSV-targets network,” it was identified that ALB, PPARG, CASP3, STAT3, TGFB1, JAK2, TLR4, PRKACA, and PRKACB might serve as core targets of MXSGS in treating PRRSV. Molecular docking analysis confirmed that all key active ingredients of MXSGS had a high binding affinity to potential core targets associated with PRRSV. PPARG, known as peroxisome proliferator-activated receptor gamma (PPAR*γ*), presents immunomodulatory and antiviral features (De Carvalho et al., 2021). The agonist of PPARγ—Telmisartan (TM), could significantly inhibit chikungunya fever virus (CHIKV) via activating AT1/PPAR-γ/MAPKs pathways (De et al., 2022). CASP3, called Caspase-3, plays a key role in the cleavage of nuclear proteins, promoting nuclear breakdown and apoptosis execution (Prokhorova et al., 2018). Wang et al. (2013) revealed that dipotassium glycyrrhizinate effectively inhibited PRRSV replication by suppressing apoptosis mediated by caspase-3. JAK2 (Janus kinase 2) and STAT3 (Signal transducer and activator of transcription 3) play crucial roles in the JAK–STAT signaling pathway mediating immune response (Samra et al., 2024). Historically, PRRSV could inhibit interferon-induced JAK–STAT signaling by obstructing nuclear translocation of STAT1/STAT2 and antagonizing IL6-mediated JAK–STAT3 signaling by accelerating STAT3 degradation (Yang and Zhang, 2017; Yang et al., 2017). Therefore, we speculated that MXSGS could also be used against PRRSV by activating the JAK/STAT signaling pathway. TGFB1, a multifunctional growth factor, inhibits macrophage activation by inhibiting IFN-γ synthesis and promoting IL-10 production (Gómez-Laguna et al., 2012). Knockdown of TGFB1 resulted in the antiviral immunity enhancement in peripheral blood mononuclear cells of Tibetan pigs (Tp-PBMCs) and significant inhibition of PRRSV proliferation (Wang et al., 2019). TLR4, a toll-like receptor, is vital in pathogen recognition and innate immunity activation (Gupta et al., 2021). Matrine possessed activity against PRRSV/PCV2 co-infection *in vitro* by suppressing the TLR3,4/NF-κB/TNF-*α* pathway (Sun et al., 2016). PRKACA/B, known as protein kinase cAMP-activated catalytic subunit alpha/beta genes, encoded catalytic protein kinase A (PKA) subunits, which negatively regulated the expression of type I interferon and downstream antiviral genes induced by RNA viruses through catalyzing phosphorylation modification of threonine at position 54 of VISA, preventing the continuous activation of signaling pathways and the occurrence of excessive immune responses (Xu et al., 2005; Yan et al., 2017). These findings suggested that the MXSGS could interact with the core targets to modulate immune responses and inflammatory processes, thereby exerting an antiviral effect on PRRSV.

Notably, this study had certain limitations in using some human-related disease databases. Nevertheless, comparative genomics showed that over 80% of genes are homologous between pigs and humans, which led to swine becoming a major mammalian model for human studies in organ development and disease progression (Lunney, 2007; Teng et al., 2024). Therefore, this biological similarity makes it reasonable to a certain extent to apply network pharmacology analysis to drug development for porcine diseases.

## 5 Conclusion

In conclusion, our studies incorporate *in vitro* experiments and network pharmacology analysis to decipher MXSGS’s practical ability to treat PRRSV. Moreover, we analyzed MXSGS’s active components and potential anti-PRRSV targets, which provided important insights for future swine disease research involving TCMs.

## Data Availability

The datasets presented in this study can be found in online repositories. The names of the repository/repositories and accession number(s) can be found in the article/Supplementary material.

## Footnotes

^1^https://old.tcmsp-e.com/tcmsp.php, accessed on 25 August 2024.

^2^https://pubchem.ncbi.nlm.nih.gov/, accessed on 29 August 2024.

^3^http://www.swissadme.ch/, accessed on 6 September 2024.

^4^http://www.swisstargetprediction.ch/, accessed on 5 September 2024.

^5^http://stitch.embl.de/, accessed on 10 September 2024.

^6^https://www.uniprot.org/, accessed on 15 September 2024.

^7^https://www.genecards.org/, accessed on 25 August 2024.

^8^https://omim.org/, accessed on 25 August 2024.

^9^https://www.pharmgkb.org/, accessed on 25 August 2024.

^10^https://cn.string-db.org/, accessed on 16 September 2024.

^11^https://david.ncifcrf.gov/, accessed on 20 September 2024.

^12^http://www.bioinformatics.com.cn/, accessed on 23 September 2024.

^13^https://www.rcsb.org/, accessed on 30 September 2024.

## References

[ref1] Bello-Onaghise G. Wang G. Han X. Nsabimana E. Cui W. Yu F.. (2020). Antiviral strategies of Chinese herbal medicine against PRRSV infection. Front. Microbiol. 11:1756. doi: 10.3389/fmicb.2020.01756, PMID: 32849384 PMC7401453

[ref2] Chang K. Fan K. Zhang H. Wu Q. Zhang Y. Wang L.. (2024). Fuzhengjiedu san inhibits porcine reproductive and respiratory syndrome virus by activating the PI3K/AKT pathway. PLoS One 19:e0283728. doi: 10.1371/journal.pone.0283728, PMID: 38709810 PMC11073700

[ref3] Chen L. Li Z. Tang Y. Cui X. Luo R. Guo S.. (2011). Isolation, identification and antiviral activities of metabolites of calycosin-7-O-β-D-glucopyranoside. J. Pharm. Biomed. Anal. 56, 382–389. doi: 10.1016/j.jpba.2011.05.033, PMID: 21703796

[ref4] Cui Z. Liu J. Xie C. Wang T. Sun P. Wang J.. (2024). High-throughput screening unveils nitazoxanide as a potent PRRSV inhibitor by targeting NMRAL1. Nat. Commun. 15:4813. doi: 10.1038/s41467-024-48807-y, PMID: 38844461 PMC11156899

[ref5] De Carvalho M. V. Gonçalves-de-Albuquerque C. F. Silva A. R. (2021). PPAR gamma: from definition to molecular targets and therapy of lung diseases. Int. J. Mol. Sci. 22:805. doi: 10.3390/ijms22020805, PMID: 33467433 PMC7830538

[ref6] De S. Mamidi P. Ghosh S. Keshry S. S. Mahish C. Pani S. S.. (2022). Telmisartan restricts chikungunya virus infection in vitro and in vivo through the AT1/PPAR-γ/MAPKs pathways. Antimicrob. Agents Chemother. 66:e0148921. doi: 10.1128/AAC.01489-21, PMID: 34748384 PMC8765259

[ref7] Deng M. Chen H. Long J. Song J. Xie L. Li X. (2021). Calycosin: a review of its pharmacological effects and application prospects. Expert Rev. Anti-Infect. Ther. 19, 911–925. doi: 10.1080/14787210.2021.1863145, PMID: 33346681

[ref8] Deng Z. Hassan S. Rafiq M. Li H. He Y. Cai Y.. (2020). Pharmacological activity of Eriodictyol: the major natural polyphenolic flavanone. Evid. Based Complement. Alternat. Med. 2020:6681352. doi: 10.1155/2020/6681352, PMID: 33414838 PMC7752289

[ref9] DennisG.ShermanB. T.HosackD. A.YangJ.GaoW.LaneH. C.. (2003). DAVID: database for annotation, visualization, and integrated discovery. Genome Biol. 4:P3. doi: 10.1186/gb-2003-4-5-p312734009

[ref10] Doncheva N. T. Morris J. H. Gorodkin J. Jensen L. J. (2019). Cytoscape StringApp: network analysis and visualization of proteomics data. J. Proteome Res. 18, 623–632. doi: 10.1021/acs.jproteome.8b00702, PMID: 30450911 PMC6800166

[ref11] Du T. Nan Y. Xiao S. Zhao Q. Zhou E.-M. (2017). Antiviral strategies against PRRSV infection. Trends Microbiol. 25, 968–979. doi: 10.1016/j.tim.2017.06.001, PMID: 28652073

[ref12] Duan E. Wang D. Fang L. Ma J. Luo J. Chen H.. (2015). Suppression of porcine reproductive and respiratory syndrome virus proliferation by glycyrrhizin. Antivir. Res. 120, 122–125. doi: 10.1016/j.antiviral.2015.06.001, PMID: 26055123 PMC7113688

[ref13] Ge M. Xiao Y. Chen H. Luo F. Du G. Zeng F. (2018). Multiple antiviral approaches of (−)-epigallocatechin-3-gallate (EGCG) against porcine reproductive and respiratory syndrome virus infection in vitro. Antivir. Res. 158, 52–62. doi: 10.1016/j.antiviral.2018.07.012, PMID: 30048655

[ref14] Gómez-Laguna J. Rodríguez-Gómez I. M. Barranco I. Pallarés F. J. Salguero F. J. Carrasco L. (2012). Enhanced expression of TGFβ protein in lymphoid organs and lung, but not in serum, of pigs infected with a European field isolate of porcine reproductive and respiratory syndrome virus. Vet. Microbiol. 158, 187–193. doi: 10.1016/j.vetmic.2012.02.003, PMID: 22397935 PMC7125780

[ref15] Guang Q. Zhang L.-Z. Tang X. Li J.-K. Cao C. Chen H.-B.. (2024). Quercetin alleviates inflammation induced by porcine reproductive and respiratory syndrome virus in MARC-145 cells through the regulation of arachidonic acid and glutamine metabolism. Vet Med Sci 10:e1536. doi: 10.1002/vms3.1536, PMID: 39016357 PMC11253185

[ref16] Gupta S. Tsoporis J. N. Jia S.-H. Dos Santos C. C. Parker T. G. Marshall J. C. (2021). Toll-like receptors, associated biochemical signaling networks, and S100 ligands. Shock 56, 167–177. doi: 10.1097/SHK.0000000000001704, PMID: 33350801

[ref17] Huang K. Zhang P. Zhang Z. Youn J. Y. Wang C. Zhang H.. (2021). Traditional Chinese medicine (TCM) in the treatment of COVID-19 and other viral infections: efficacies and mechanisms. Pharmacol. Ther. 225:107843. doi: 10.1016/j.pharmthera.2021.107843, PMID: 33811957 PMC8011334

[ref18] Ji L. Song T. Ge C. Wu Q. Ma L. Chen X.. (2023). Identification of bioactive compounds and potential mechanisms of scutellariae radix-coptidis rhizoma in the treatment of atherosclerosis by integrating network pharmacology and experimental validation. Biomed. Pharmacother. 165:115210. doi: 10.1016/j.biopha.2023.115210, PMID: 37499457

[ref19] Khatun A. Shabir N. Seo B.-J. Kim B.-S. Yoon K.-J. Kim W.-I. (2016). The attenuation phenotype of a ribavirin-resistant porcine reproductive and respiratory syndrome virus is maintained during sequential passages in pigs. J. Virol. 90, 4454–4468. doi: 10.1128/JVI.02836-15, PMID: 26889041 PMC4836337

[ref20] Li Y. Chu F. Li P. Johnson N. Li T. Wang Y.. (2021). Potential effect of maxing Shigan decoction against coronavirus disease 2019 (COVID-19) revealed by network pharmacology and experimental verification. J. Ethnopharmacol. 271:113854. doi: 10.1016/j.jep.2021.113854, PMID: 33513419 PMC7835541

[ref21] Li C. Fan A. Liu Z. Wang G. Zhou L. Zhang H.. (2024). Prevalence, time of infection, and diversity of porcine reproductive and respiratory syndrome virus in China. Viruses 16:774. doi: 10.3390/v16050774, PMID: 38793655 PMC11125865

[ref22] Li J. Miller L. C. Sang Y. (2024). Current status of vaccines for porcine reproductive and respiratory syndrome: interferon response, immunological overview, and future prospects. Vaccines 12:606. doi: 10.3390/vaccines12060606, PMID: 38932335 PMC11209547

[ref23] Li P. Shen Y. Wang T. Li J. Li Y. Zhao Y.. (2022). Epidemiological survey of PRRS and genetic variation analysis of the ORF5 gene in Shandong Province, 2020-2021. Front. Vet. Sci. 9:987667. doi: 10.3389/fvets.2022.987667, PMID: 36187820 PMC9521713

[ref24] Liu Y. Zhang J.-T. Sun M. Song J. Sun H.-M. Wang M.-Y.. (2025). Targeting ferroptosis in the treatment of ulcerative colitis by traditional Chinese medicine: a novel therapeutic strategies. Phytomedicine 139:156539. doi: 10.1016/j.phymed.2025.156539, PMID: 39987602

[ref25] Liu Y. Zhang L. Xue B. Chen L. Wang G. Wang J.. (2024). Simulation of red mud/phosphogypsum-based artificial soil engineering applications in vegetation restoration and ecological reconstruction. Sci. Total Environ. 951:175656. doi: 10.1016/j.scitotenv.2024.175656, PMID: 39168339

[ref26] Lunney J. K. (2007). Advances in swine biomedical model genomics. Int. J. Biol. Sci. 3, 179–184. doi: 10.7150/ijbs.3.179, PMID: 17384736 PMC1802015

[ref27] Paggi J. M. Pandit A. Dror R. O. (2024). The art and science of molecular docking. Annu. Rev. Biochem. 93, 389–410. doi: 10.1146/annurev-biochem-030222-120000, PMID: 38594926 PMC13198409

[ref28] Pizzi M. (1950). Sampling variation of the fifty percent end-point, determined by the reed-Muench (Behrens) method. Hum. Biol. 22, 151–190, PMID: 14778593

[ref29] Prokhorova E. A. Kopeina G. S. Lavrik I. N. Zhivotovsky B. (2018). Apoptosis regulation by subcellular relocation of caspases. Sci. Rep. 8:12199. doi: 10.1038/s41598-018-30652-x, PMID: 30111833 PMC6093910

[ref30] Ru J. Li P. Wang J. Zhou W. Li B. Huang C.. (2014). TCMSP: a database of systems pharmacology for drug discovery from herbal medicines. J. Cheminformatics 6:13. doi: 10.1186/1758-2946-6-13, PMID: 24735618 PMC4001360

[ref31] Samra S. Bergerson J. R. E. Freeman A. F. Turvey S. E. (2024). Inborn errors of immunity and somatic variant phenocopies disrupting human JAK-STAT signaling. J. Allergy Clin. Immunol. 155, 357–367. doi: 10.1016/j.jaci.2024.09.020, PMID: 39369964

[ref32] Sang Y. Rowland R. R. Blecha F. (2011). Porcine type I interferons: polymorphic sequences and activity against PRRSV. BMC Proc. 5:S8. doi: 10.1186/1753-6561-5-S4-S8, PMID: 21645323 PMC3108238

[ref33] Shannon P. Markiel A. Ozier O. Baliga N. S. Wang J. T. Ramage D.. (2003). Cytoscape: a software environment for integrated models of biomolecular interaction networks. Genome Res. 13, 2498–2504. doi: 10.1101/gr.1239303, PMID: 14597658 PMC403769

[ref34] Sun N. Sun P. Lv H. Sun Y. Guo J. Wang Z.. (2016). Matrine displayed antiviral activity in porcine alveolar macrophages co-infected by porcine reproductive and respiratory syndrome virus and porcine circovirus type 2. Sci. Rep. 6:24401. doi: 10.1038/srep24401, PMID: 27080155 PMC4832146

[ref35] Sun Y. Xing J. Hong S. L. Bollen N. Xu S. Li Y.. (2024). Untangling lineage introductions, persistence and transmission drivers of HP-PRRSV sublineage 8.7. Nat. Commun. 15:8842. doi: 10.1038/s41467-024-53076-w, PMID: 39397015 PMC11471759

[ref36] Teng J. Gao Y. Yin H. Bai Z. Liu S. Zeng H.. (2024). A compendium of genetic regulatory effects across pig tissues. Nat. Genet. 56, 112–123. doi: 10.1038/s41588-023-01585-7, PMID: 38177344 PMC10786720

[ref37] Theoharides T. C. Kempuraj D. (2023). Role of SARS-CoV-2 spike-protein-induced activation of microglia and mast cells in the pathogenesis of neuro-COVID. Cells 12:688. doi: 10.3390/cells12050688, PMID: 36899824 PMC10001285

[ref38] Wang C. Cao B. Liu Q.-Q. Zou Z.-Q. Liang Z.-A. Gu L.. (2011). Oseltamivir compared with the Chinese traditional therapy maxingshigan-yinqiaosan in the treatment of H1N1 influenza: a randomized trial. Ann. Intern. Med. 155, 217–225. doi: 10.7326/0003-4819-155-4-201108160-00005, PMID: 21844547

[ref39] Wang Y. Chen Y. Liang G. Zeng K. Chen X.-H. Ying S.-C.. (2019). Silence of TGF-β1 gene expression reduces prrsv replication and potentiates immunity of immune cells of tibetan pig. Vet. Anim. Sci. 8:100074. doi: 10.1016/j.vas.2019.100074, PMID: 32734091 PMC7386707

[ref40] Wang Z.-W. Sun N. Wu C.-H. Jiang J.-B. Bai Y.-S. Li H.-Q. (2013). In vitro antiviral activity and underlying molecular mechanisms of dipotassium glycyrrhetate against porcine reproductive and respiratory syndrome virus. Antivir. Ther. 18, 997–1004. doi: 10.3851/IMP2662, PMID: 23872789

[ref41] Xu L.-G. Wang Y.-Y. Han K.-J. Li L.-Y. Zhai Z. Shu H.-B. (2005). VISA is an adapter protein required for virus-triggered IFN-beta signaling. Mol. Cell 19, 727–740. doi: 10.1016/j.molcel.2005.08.014, PMID: 16153868

[ref42] Yan B.-R. Zhou L. Hu M.-M. Li M. Lin H. Yang Y.. (2017). PKACs attenuate innate antiviral response by phosphorylating VISA and priming it for MARCH5-mediated degradation. PLoS Pathog. 13:e1006648. doi: 10.1371/journal.ppat.1006648, PMID: 28934360 PMC5626498

[ref43] Yang Y. Liu Y. Lou R. Lei Y. Li G. Xu Z.. (2023). Glycyrrhiza polysaccharides inhibits PRRSV replication. Virol. J. 20:140. doi: 10.1186/s12985-023-02052-9, PMID: 37408066 PMC10320881

[ref44] Yang L. Wang R. Ma Z. Xiao Y. Nan Y. Wang Y.. (2017). Porcine reproductive and respiratory syndrome virus antagonizes JAK/STAT3 signaling via nsp5, which induces STAT3 degradation. J. Virol. 91, e02087–e02016. doi: 10.1128/JVI.02087-16, PMID: 27881658 PMC5244345

[ref45] Yang L. Zhang Y.-J. (2017). Antagonizing cytokine-mediated JAK-STAT signaling by porcine reproductive and respiratory syndrome virus. Vet. Microbiol. 209, 57–65. doi: 10.1016/j.vetmic.2016.12.036, PMID: 28069291 PMC7117332

[ref46] Ye C. Gao Z. H. Bie Z.-Y. Chen K.-Q. Lu F. G. Wei K. (2023). MXSGD alleviates CsA-induced hypoimmunity lung injury by regulating microflora metabolism. Front. Immunol. 14:1298416. doi: 10.3389/fimmu.2023.1298416, PMID: 38259457 PMC10801022

[ref47] Yin H. Li Y. Feng Y. Tian L. Li Y. (2024). The extraction, biosynthesis, health-promoting and therapeutic properties of natural flavanone Eriodictyol. Nutrients 16:4237. doi: 10.3390/nu16234237, PMID: 39683630 PMC11644446

[ref48] Yu Z. Wu Z. Wang Z. Wang Y. Zhou M. Li W.. (2024). Network-based methods and their applications in drug discovery. J. Chem. Inf. Model. 64, 57–75. doi: 10.1021/acs.jcim.3c01613, PMID: 38150548

[ref49] Zhang F.-L. Chen Y.-L. Luo Z.-Y. Song Z.-B. Chen Z. Zhang J.-X.. (2025). Huashi baidu granule alleviates inflammation and lung edema by suppressing the NLRP3/caspase-1/GSDMD-N pathway and promoting fluid clearance in a porcine reproductive and respiratory syndrome (PRRS) model. J. Ethnopharmacol. 340:119207. doi: 10.1016/j.jep.2024.119207, PMID: 39653102

[ref50] Zhang M.-L. Irwin D. Li X.-N. Sauriol F. Shi X.-W. Wang Y.-F.. (2012). PPARγ agonist from *Chromolaena odorata*. J. Nat. Prod. 75, 2076–2081. doi: 10.1021/np300386d, PMID: 23186307

[ref51] Zhang Z.-H. Yuan C.-Y. Xu M. Wang M.-F. Feng T. Wang Y.. (2024). Calycosin inhibits the proliferation and metastasis of renal cell carcinoma through the MAZ/HAS2 signaling pathway. Phytother. Res. 38, 4774–4791. doi: 10.1002/ptr.8295, PMID: 39120474

