# Supplementary material for: Antiviral effects and mechanism of Ma-Xing-Shi-Gan-San on porcine reproductive and respiratory syndrome virus
Source: Front Microbiol. 2025 Apr 29;16:1539094. doi: 10.3389/fmicb.2025.1539094 (PMC12069324; doi:10.3389/fmicb.2025.1539094)
Supplement: Supplementary file 1 [file Table_1.DOCX]

Table S1 The active ingredients of MXSGS obtained through the TCMSP database and literature of predecessors

| **Component** | **Network  Number** | **Molecule Name** | **OB (%)** | **DL** | **SMILES** |
| --- | --- | --- | --- | --- | --- |
| Mahuang | M1 | Diosmetin | 31.14 | 0.27 | COC1=C(C=C(C=C1)C2=CC(=O)C3=C(C=C(C=C3O2)O)O)O |
|  | M2 | eriodictyol | 71.79 | 0.24 | C1[C@H](OC2=CC(=CC(=C2C1=O)O)O)C3=CC(=C(C=C3)O)O |
|  | M3 | Genkwanin | 37.13 | 0.24 | COC1=CC(=C2C(=C1)OC(=CC2=O)C3=CC=C(C=C3)O)O |
|  | M4 | Herbacetin | 36.07 | 0.27 | C1=CC(=CC=C1C2=C(C(=O)C3=C(O2)C(=C(C=C3O)O)O)O)O |
|  | MG1 | kaempferol | 41.88 | 0.24 | C1=CC(=CC=C1C2=C(C(=O)C3=C(C=C(C=C3O2)O)O)O)O |
|  | M6 | luteolin | 36.16 | 0.25 | C1=CC(=C(C=C1C2=CC(=O)C3=C(C=C(C=C3O2)O)O)O)O |
|  | MG2 | naringenin | 59.29 | 0.21 | C1[C@H](OC2=CC(=CC(=C2C1=O)O)O)C3=CC=C(C=C3)O |
|  | M8 | Pectolinarigenin | 41.17 | 0.3 | COC1=CC=C(C=C1)C2=CC(=O)C3=C(O2)C=C(C(=C3O)OC)O |
|  | MG3 | quercetin | 46.43 | 0.28 | C1=CC(=C(C=C1C2=C(C(=O)C3=C(C=C(C=C3O2)O)O)O)O)O |
|  | M10 | Truflex OBP | 43.74 | 0.24 | CCCCCCCCOC(=O)C1=CC=CC=C1C(=O)OCCCC |
| Xingren | X1 | estrone | 53.56 | 0.32 | C[C@]12CC[C@H]3[C@H]([C@@H]1CCC2=O)CCC4=C3C=CC(=C4)O |
|  | XG1 | Glabridin | 53.25 | 0.47 | CC1(C=CC2=C(O1)C=CC3=C2OC[C@H](C3)C4=C(C=C(C=C4)O)O)C |
|  | XG2 | Licochalcone B | 76.76 | 0.19 | COC1=C(C=CC(=C1O)O)/C=C/C(=O)C2=CC=C(C=C2)O |
|  | X4 | l-SPD | 87.35 | 0.54 | COC1=C(C=C2[C@@H]3CC4=C(CN3CCC2=C1)C(=C(C=C4)O)OC)O |
|  | X5 | Machiline | 79.64 | 0.24 | COC1=C(C=C2C(NCCC2=C1)CC3=CC=C(C=C3)O)O |
|  | XG3 | Phaseol | 78.77 | 0.58 | CC(=CCC1=C(C=CC2=C1OC(=O)C3=C2OC4=C3C=CC(=C4)O)O)C |
| Gancao | G1 | (2R)-7-hydroxy-2-(4-hydroxyphenyl)chroman-4-one | 71.12 | 0.18 | C1[C@@H](OC2=C(C1=O)C=CC(=C2)O)C3=CC=C(C=C3)O |
|  | G2 | (2S)-2-[4-hydroxy-3-(3-methylbut-2-enyl)phenyl]-8,8-dimethyl-2,3-dihydropyrano[2,3-f]chromen-4-one | 31.79 | 0.72 | CC(=CCC1=C(C=CC(=C1)[C@@H]2CC(=O)C3=C(O2)C4=C(C=C3)OC(C=C4)(C)C)O)C |
|  | G3 | (2S)-6-(2,4-dihydroxyphenyl)-2-(2-hydroxypropan-2-yl)-4-methoxy-2,3-dihydrofuro[3,2-g]chromen-7-one | 60.25 | 0.63 | CC(C)([C@@H]1CC2=C(O1)C=C3C(=C2OC)C=C(C(=O)O3)C4=C(C=C(C=C4)O)O)O |
|  | G4 | (2S)-7-hydroxy-2-(4-hydroxyphenyl)-8-(3-methylbut-2-enyl)chroman-4-one | 36.57 | 0.32 | CC(C)=CCc1c(O)ccc2c1O[C@H](c1ccc(O)cc1)CC2=O |
|  | G5 | (E)-1-(2,4-dihydroxyphenyl)-3-(2,2-dimethylchromen-6-yl)prop-2-en-1-one | 39.62 | 0.35 | CC1(C=CC2=C(O1)C=CC(=C2)/C=C/C(=O)C3=C(C=C(C=C3)O)O)C |
|  | G6 | (E)-3-[3,4-dihydroxy-5-(3-methylbut-2-enyl)phenyl]-1-(2,4-dihydroxyphenyl)prop-2-en-1-one | 46.27 | 0.31 | CC(=CCC1=C(C(=CC(=C1)/C=C/C(=O)C2=C(C=C(C=C2)O)O)O)O)C |
|  | G7 | 1,3-dihydroxy-8,9-dimethoxy-6-benzofurano[3,2-c]chromenone | 62.9 | 0.53 | COc1cc2oc3c4c(O)cc(O)cc4oc(=O)c3c2cc1OC |
|  | G8 | 1,3-dihydroxy-9-methoxy-6-benzofurano[3,2-c]chromenone | 48.14 | 0.43 | COc1ccc2c(c1)oc1c3c(O)cc(O)cc3oc(=O)c21 |
|  | G9 | 1-Methoxyphaseollidin | 69.98 | 0.64 | CC(=CCC1=C(C=CC2=C1O[C@@H]3[C@H]2COC4=C3C(=CC(=C4)O)OC)O)C |
|  | G10 | 2-(3,4-dihydroxyphenyl)-5,7-dihydroxy-6-(3-methylbut-2-enyl)chromone | 44.15 | 0.41 | CC(C)=CCc1c(O)cc2oc(-c3ccc(O)c(O)c3)cc(=O)c2c1O |
|  | G11 | 2-[(3R)-8,8-dimethyl-3,4-dihydro-2H-pyrano[6,5-f]chromen-3-yl]-5-methoxyphenol | 36.21 | 0.52 | COc1ccc([C@@H]2COc3c(ccc4c3C=CC(C)(C)O4)C2)c(O)c1 |
|  | G12 | 3-(3,4-dihydroxyphenyl)-5,7-dihydroxy-8-(3-methylbut-2-enyl)chromone | 66.37 | 0.41 | CC(C)=CCc1c(O)cc(O)c2c(=O)c(-c3ccc(O)c(O)c3)coc12 |
|  | G13 | 3'-Hydroxy-4'-O-Methylglabridin | 43.71 | 0.57 | CC1(C=CC2=C(O1)C=CC3=C2OCC(C3)C4=C(C(=C(C=C4)OC)O)O)C |
|  | G14 | 3'-Methoxyglabridin | 46.16 | 0.57 | CC1(C=CC2=C(O1)C=CC3=C2OCC(C3)C4=C(C(=C(C=C4)O)OC)O)C |
|  | G15 | 6-prenylated eriodictyol | 39.22 | 0.41 | CC(C)=CCc1c(O)cc2c(c1O)C(=O)C[C@H](c1ccc(O)c(O)c1)O2 |
|  | G16 | 7,2',4'-trihydroxy－5-methoxy-3－arylcoumarin | 83.71 | 0.27 | COC1=CC(=CC2=C1C=C(C(=O)O2)C3=C(C=C(C=C3)O)O)O |
|  | G17 | 7-Acetoxy-2-methylisoflavone | 38.92 | 0.26 | CC1=C(C(=O)C2=C(O1)C=C(C=C2)OC(=O)C)C3=CC=CC=C3 |
|  | G18 | 7-Methoxy-2-methyl isoflavone | 42.56 | 0.2 | CC1=C(C(=O)C2=C(O1)C=C(C=C2)OC)C3=CC=C(C=C3)[N+](=O)[O-] |
|  | G19 | 8-(6-hydroxy-2-benzofuranyl)-2,2-dimethyl-5-chromenol | 58.44 | 0.38 | CC1(C)C=Cc2c(O)ccc(-c3cc4ccc(O)cc4o3)c2O1 |
|  | G20 | 8-prenylated eriodictyol | 53.79 | 0.4 | CC(C)=CCc1c(O)cc(O)c2c1O[C@@H](c1ccc(O)c(O)c1)CC2=O |
|  | G21 | Calycosin | 47.75 | 0.24 | COC1=C(C=C(C=C1)C2=COC3=C(C2=O)C=CC(=C3)O)O |
|  | G22 | DFV | 32.76 | 0.18 | C1[C@H](OC2=C(C1=O)C=CC(=C2)O)C3=CC=C(C=C3)O |
|  | G23 | euchrenone | 30.29 | 0.57 | CC(=CCC1=C(C=CC2=C1O[C@@H](CC2=O)C3=C(C=C(C=C3)O)O)O)C |
|  | G24 | Eurycarpin A | 43.28 | 0.37 | CC(=CCC1=C(C=CC(=C1O)C2=COC3=C(C2=O)C=CC(=C3)O)O)C |
|  | G25 | formononetin | 69.67 | 0.21 | COC1=CC=C(C=C1)C2=COC3=C(C2=O)C=CC(=C3)O |
|  | G26 | Glabranin | 52.9 | 0.31 | CC(=CCC1=C2C(=C(C=C1O)O)C(=O)C[C@H](O2)C3=CC=CC=C3)C |
|  | G27 | Glabrene | 46.27 | 0.44 | CC1(C=CC2=C(C=CC(=C2O1)C3=CC4=C(C=C(C=C4)O)OC3)O)C |
|  | XG1 | Glabridin | 53.25 | 0.47 | CC1(C=CC2=C(O1)C=CC3=C2OC[C@H](C3)C4=C(C=C(C=C4)O)O)C |
|  | G29 | Glabrone | 52.51 | 0.5 | CC1(C=CC2=C(O1)C=CC(=C2O)C3=COC4=C(C3=O)C=CC(=C4)O)C |
|  | G30 | glyasperin B | 65.22 | 0.44 | CC(=CCC1=C(C=C2C(=C1O)C(=O)C(CO2)C3=C(C=C(C=C3)O)O)OC)C |
|  | G31 | Glyasperin C | 45.56 | 0.4 | CC(=CCC1=C(C2=C(C=C1O)OC[C@H](C2)C3=C(C=C(C=C3)O)O)OC)C |
|  | G32 | glyasperin F | 75.84 | 0.54 | CC1(C=CC2=C(C=CC(=C2O1)C3COC4=CC(=CC(=C4C3=O)O)O)O)C |
|  | G33 | Glyasperins M | 72.67 | 0.59 | COc1cc(O)cc2c1C(=O)[C@H](c1ccc3c(c1O)C=CC(C)(C)O3)CO2 |
|  | G34 | Glypallichalcone | 61.6 | 0.19 | COC1=CC=C(C=C1)C(=O)/C=C/C2=C(C=C(C=C2)O)OC |
|  | G35 | Glyzaglabrin | 61.07 | 0.35 | C1OC2=C(O1)C(=C(C=C2)C3=COC4=C(C3=O)C=CC(=C4)O)O |
|  | G36 | HMO | 38.37 | 0.21 | COC1=CC2=C(C=C1)C(=O)C(=CO2)C3=CC=C(C=C3)O |
|  | G37 | Inermine | 75.18 | 0.54 | C1[C@@H]2[C@H](C3=C(O1)C=C(C=C3)O)OC4=CC5=C(C=C24)OCO5 |
|  | G38 | Inflacoumarin A | 39.71 | 0.33 | CC(C)=CCc1cc2c(-c3ccc(O)cc3)cc(=O)oc2cc1O |
|  | G39 | Isoglycyrol | 44.7 | 0.84 | CC1(CCC2=C(O1)C=C3C(=C2OC)C4=C(C5=C(O4)C=C(C=C5)O)C(=O)O3)C |
|  | G40 | Isolicoflavonol | 45.17 | 0.42 | CC(=CCC1=C(C=CC(=C1)C2=C(C(=O)C3=C(C=C(C=C3O2)O)O)O)O)C |
|  | G41 | isorhamnetin | 49.6 | 0.31 | COC1=C(C=CC(=C1)C2=C(C(=O)C3=C(C=C(C=C3O2)O)O)O)O |
|  | G42 | Isotrifoliol | 31.94 | 0.42 | COC1=CC(=CC2=C1C3=C(C4=C(O3)C=C(C=C4)O)C(=O)O2)O |
|  | G43 | Jaranol | 50.83 | 0.29 | COC1=CC(=C2C(=C1)OC(=C(C2=O)OC)C3=CC=C(C=C3)O)O |
|  | MG1 | kaempferol | 41.88 | 0.24 | C1=CC(=CC=C1C2=C(C(=O)C3=C(C=C(C=C3O2)O)O)O)O |
|  | G45 | Kanzonol F | 32.47 | 0.89 | CC(=CCC1=C(C2=C(C=C1O)OC[C@@H]3[C@H]2OC4=C3C=C5C=CC(OC5=C4)(C)C)OC)C |
|  | G46 | Licoagrocarpin | 58.81 | 0.58 | CC(=CCC1=C(C=CC2=C1OC[C@@H]3[C@H]2OC4=C3C=CC(=C4)OC)O)C |
|  | G47 | Licoagroisoflavone | 57.28 | 0.49 | CC(=C)[C@H]1CC2=C(O1)C=C3C(=C2O)C(=O)C(=CO3)C4=CC=C(C=C4)O |
|  | G48 | licochalcone a | 40.79 | 0.29 | CC(C)(C=C)C1=C(C=C(C(=C1)/C=C/C(=O)C2=CC=C(C=C2)O)OC)O |
|  | XG2 | Licochalcone B | 76.76 | 0.19 | COC1=C(C=CC(=C1O)O)/C=C/C(=O)C2=CC=C(C=C2)O |
|  | G50 | licochalcone G | 49.25 | 0.32 | CC(C)(C=C)C1=C(C=C(C(=C1)/C=C/C(=O)C2=C(C=C(C=C2)O)O)OC)O |
|  | G51 | Licocoumarone | 33.21 | 0.36 | CC(=CCC1=C(C2=C(C=C1O)OC(=C2)C3=C(C=C(C=C3)O)O)OC)C |
|  | G52 | licoisoflavanone | 52.47 | 0.54 | CC1(C=CC2=C(O1)C=CC(=C2O)C3COC4=CC(=CC(=C4C3=O)O)O)C |
|  | G53 | Licoisoflavone | 41.61 | 0.42 | CC(=CCC1=C(C=CC(=C1O)C2=COC3=CC(=CC(=C3C2=O)O)O)O)C |
|  | G54 | Licoisoflavone B | 38.93 | 0.55 | CC1(C=CC2=C(O1)C=CC(=C2O)C3=COC4=CC(=CC(=C4C3=O)O)O)C |
|  | G55 | licopyranocoumarin | 80.36 | 0.65 | CC1(CCC2=C(O1)C=C3C(=C2OC)C=C(C(=O)O3)C4=C(C=C(C=C4)O)O)CO |
|  | G56 | Medicarpin | 49.22 | 0.34 | COC1=CC2=C(C=C1)[C@@H]3COC4=C([C@@H]3O2)C=CC(=C4)O |
|  | MG2 | naringenin | 59.29 | 0.21 | C1[C@H](OC2=CC(=CC(=C2C1=O)O)O)C3=CC=C(C=C3)O |
|  | G58 | Odoratin | 49.95 | 0.3 | COC1=C(C=C(C=C1)C2=COC3=CC(=C(C=C3C2=O)OC)O)O |
|  | XG3 | Phaseol | 78.77 | 0.58 | CC(=CCC1=C(C=CC2=C1OC(=O)C3=C2OC4=C3C=CC(=C4)O)O)C |
|  | G60 | Phaseolinisoflavan | 32.01 | 0.45 | CC1(C=CC2=C(O1)C=CC(=C2O)[C@H]3CC4=C(C=C(C=C4)O)OC3)C |
|  | MG3 | quercetin | 46.43 | 0.28 | C1=CC(=C(C=C1C2=C(C(=O)C3=C(C=C(C=C3O2)O)O)O)O)O |
|  | G62 | Quercetin der. | 46.45 | 0.33 | COc1cc(-c2oc3cc(O)cc(O)c3c(=O)c2OC)ccc1O |
|  | G63 | Semilicoisoflavone B | 48.78 | 0.55 | CC1(C=CC2=C(O1)C(=CC(=C2)C3=COC4=CC(=CC(=C4C3=O)O)O)O)C |
|  | G64 | shinpterocarpin | 80.3 | 0.73 | CC1(C=CC2=C(O1)C=CC3=C2OC[C@@H]4[C@H]3OC5=C4C=CC(=C5)O)C |
|  | G65 | Sigmoidin-B | 34.88 | 0.41 | CC(=CCC1=C(C(=CC(=C1)[C@@H]2CC(=O)C3=C(C=C(C=C3O2)O)O)O)O)C |
|  | G66 | Vestitol | 74.66 | 0.21 | COC1=CC(=C(C=C1)C2CC3=C(C=C(C=C3)O)OC2)O |
|  | G67 | Xambioona | 54.85 | 0.87 | CC1(C=CC2=C(O1)C=CC(=C2)C3CC(=O)C4=C(O3)C5=C(C=C4)OC(C=C5)(C)C)C |
| Shigao | S1 | calcium（Ca） |  |  |  |
|  | S2 | iron (Fe) |  |  |  |
|  | S3 | magnesium (Mg) |  |  |  |
|  | S4 | potassium (K) |  |  |  |
|  | S5 | sodium (Na) |  |  |  |
