# Supplementary material for: Antiviral effects and mechanism of Ma-Xing-Shi-Gan-San on porcine reproductive and respiratory syndrome virus
Source: Front Microbiol. 2025 Apr 29;16:1539094. doi: 10.3389/fmicb.2025.1539094 (PMC12069324; doi:10.3389/fmicb.2025.1539094)
Supplement: Supplementary file 2 [file Table_2.DOCX]

Table S2 The targets of MXSGS predicted by TCMSP and STITCH database

| **Serial number** | **Targets** | **Serial number** | **Targets** |
| --- | --- | --- | --- |
|  | HTR2B |  | APP |
|  | AR |  | ABCG2 |
|  | CYP19A1 |  | NOS2 |
|  | ESR1 |  | SNCA |
|  | ESR2 |  | MAOA |
|  | NR3C1 |  | CXCR4 |
|  | IGF1R |  | BCL2L1 |
|  | ALOX15 |  | CAPN1 |
|  | SLC22A2 |  | CDK4 |
|  | CFTR |  | PGD |
|  | ADRB3 |  | PLAU |
|  | ROCK2 |  | CA3 |
|  | OPRM1 |  | IGFBP3 |
|  | CYP17A1 |  | PLAT |
|  | MIF |  | THRA |
|  | FYN |  | SLC9A1 |
|  | AURKB |  | DRD1 |
|  | MET |  | ADRA1D |
|  | NEK6 |  | ADRB2 |
|  | CBR1 |  | ADRB1 |
|  | OPRD1 |  | HTR2A |
|  | CYP11B1 |  | KCNN3 |
|  | PSEN2 |  | MTNR1A |
|  | TTR |  | ADRA2A |
|  | ALDH2 |  | BCHE |
|  | AKR1B1 |  | JUN |
|  | DHFR |  | HCRTR2 |
|  | HSP90AA1 |  | HCRTR1 |
|  | MMP1 |  | PIK3CG |
|  | GCGR |  | SERPINE1 |
|  | TLR9 |  | KCNH2 |
|  | AURKA |  | JAK2 |
|  | MAOB |  | CDC42 |
|  | ADAM17 |  | ADAM10 |
|  | MMP14 |  | HSP90B1 |
|  | EPHX2 |  | AVPR2 |
|  | SCD |  | F2 |
|  | TNNC1 |  | PLG |
|  | COMT |  | PRKAA1 |
|  | TGFBR1 |  | KCNMA1 |
|  | PLA2G1B |  | PTAFR |
|  | NPY5R |  | FBP1 |
|  | IDH1 |  | PRKACA |
|  | VCP |  | RYR1 |
|  | DPP4 |  | CAMK2D |
|  | ALOX5AP |  | PLCZ |
|  | CASQ2 |  | ATP4B |
|  | SCIN |  | TBXAS1 |
|  | PLCD4 |  | IL2 |
|  | SPARC |  | HSPA1A |
|  | RGN |  | PPIA |
|  | CAPZB |  | CDC25C |
|  | C1QA |  | ATM |
|  | ALB |  | CTSL |
|  | F9 |  | GUSB |
|  | VIL1 |  | TNF |
|  | CAPZA2 |  | IGFBP4 |
|  | CACNG1 |  | IGFBP5 |
|  | STEAP1 |  | IGFBP2 |
|  | TF |  | IGFBP1 |
|  | NRAMP1 |  | KDM5C |
|  | LTF |  | EPHX1 |
|  | CNN1 |  | TGFB1 |
|  | CNN2 |  | NR3C2 |
|  | PROC |  | PGK1 |
|  | ATP4A |  | BCAT2 |
|  | ATP1B1 |  | PNMT |
|  | CTSK |  | GSTP1 |
|  | ENSA |  | CHRM1 |
|  | ATP1B4 |  | BDKRB2 |
|  | SLC5A1 |  | MME |
|  | SLC4A4 |  | CPB1 |
|  | SLC2A2 |  | AGTR1 |
|  | SLC52A2 |  | TLR4 |
|  | ARG1 |  | EDNRB |
|  | SYK |  | LDLR |
|  | AKR1A1 |  | STAT3 |
|  | PREP |  | CFD |
|  | FAAH |  | ANPEP |
|  | TSPO |  | NPY1R |
|  | NAMPT |  | CCKBR |
|  | OXTR |  | GRK7 |
|  | SLC2A3 |  | BIRC5 |
|  | PPARG |  | HTR1B |
|  | EDNRA |  | PRKACB |
|  | CTSB |  | HTR1D |
|  | PTGER3 |  | HTR4 |
|  | VEGFA |  | ACE |
|  | TOP2A |  | CALCRL |
|  | MYLK |  | SRD5A2 |
|  | DAO |  | SSTR2 |
|  | CTSD |  | CASR |
|  | CYSLTR1 |  | HMGCR |
|  | CHRM3 |  | CHRM2 |
|  | NOS3 |  | PIK3C3 |
|  | CASP3 |  | ABAT |
|  | NR5A1 |  | SORD |
|  | ME1 |  | CSNK1A1 |
|  | GNRHR |  | AAK1 |
|  | ACP1 |  | KCNJ5 |
|  | CYSLTR2 |  | BDKRB1 |
|  | CALCA |  | OPRL1 |
