# Supplementary material for: Antiviral effects and mechanism of Ma-Xing-Shi-Gan-San on porcine reproductive and respiratory syndrome virus
Source: Front Microbiol. 2025 Apr 29;16:1539094. doi: 10.3389/fmicb.2025.1539094 (PMC12069324; doi:10.3389/fmicb.2025.1539094)
Supplement: Supplementary file 3 [file Table_3.DOCX]

Table S3 The targets of PRRSV obtained from GeneCards, PharmGKB, and OMIM databases

| Serial number | PRRSV Targets | Serial number | PRRSV Targets |
| --- | --- | --- | --- |
|  | TP53 |  | TGFB3 |
|  | FBN1 |  | ESR1 |
|  | WT1 |  | FASLG |
|  | SDHA |  | CAV3 |
|  | KCNH2 |  | LRP2 |
|  | SMAD4 |  | TTR |
|  | SDHB |  | ADA2 |
|  | STAT3 |  | GNRH1 |
|  | SDHD |  | CYP19A1 |
|  | MYO7A |  | STAT5B |
|  | MET |  | SERPINA1 |
|  | LMNA |  | CD46 |
|  | RYR1 |  | GJA1 |
|  | TNF |  | IGF2 |
|  | SDHC |  | ATP1A2 |
|  | INS |  | SOX9 |
|  | IL6 |  | F5 |
|  | CFTR |  | KCNMA1 |
|  | CHAT |  | AMH |
|  | FAS |  | TLR4 |
|  | MT-CYB |  | BDNF |
|  | ACTB |  | APOE |
|  | MT-CO1 |  | EDN1 |
|  | CD40LG |  | PSMB8 |
|  | TNFRSF1A |  | POR |
|  | EPCAM |  | CP |
|  | KCNJ2 |  | COMT |
|  | IL10 |  | GATM |
|  | TGFB1 |  | FOXL2 |
|  | LEP |  | PPARG |
|  | CASR |  | KCNJ5 |
|  | MT-ND4 |  | DHFR |
|  | TGFBR1 |  | GHR |
|  | SMAD3 |  | EDN3 |
|  | IL1B |  | MYH7 |
|  | TGFB2 |  | F2 |
|  | MT-CO2 |  | DES |
|  | CTLA4 |  | CYP17A1 |
|  | SFTPB |  | GBA1 |
|  | ALB |  | IL4 |
|  | CXCL8 |  | FSHR |
|  | IFNG |  | CCL2 |
|  | ACE |  | NR5A1 |
|  | C3 |  | IL1RN |
|  | SFTPC |  | IDH1 |
|  | CRP |  | GALNS |
|  | SOD1 |  | CSF3 |
|  | EDNRB |  | GLUD1 |
|  | VEGFA |  | RBP4 |
|  | MYC |  | HADHA |
|  | PSAP |  | KITLG |
|  | MYLK |  | NFKBIA |
|  | PDHA1 |  | OXTR |
|  | SRY |  | IL5 |
|  | ENG |  | ACADM |
|  | CAV1 |  | IL18 |
|  | DLD |  | DAG1 |
|  | PRL |  | CCL5 |
|  | IGF1R |  | STAR |
|  | EGF |  | VCP |
|  | IL13 |  | GPT |
|  | PLG |  | RPL11 |
|  | HMOX1 |  | GSN |
|  | IL2RA |  | TF |
|  | CFB |  | LHCGR |
|  | HBB |  | TFAM |
|  | CSF2 |  | NOS3 |
|  | LEPR |  | SFTPD |
|  | CDC42 |  | CYBB |
|  | SERPINE1 |  | GAD1 |
|  | RIGI |  | PLP1 |
|  | IDH2 |  | LOX |
|  | IL2 |  | CYCS |
|  | PIK3CG |  | GNAQ |
|  | NEU1 |  | TUBA1A |
|  | SLC2A2 |  | OTC |
|  | APOA1 |  | BGN |
|  | PRNP |  | LPL |
|  | ETFDH |  | NGF |
|  | CD40 |  | GHRL |
|  | MBL2 |  | CASP3 |
|  | GNRHR |  | SNCA |
|  | IL17A |  | MYOD1 |
|  | GH1 |  | CTSD |
|  | SFTPA1 |  | STAT2 |
|  | GMPPB |  | NPPA |
|  | NR3C1 |  | ETFB |
|  | VWF |  | NPC2 |
|  | POMC |  | B2M |
|  | CYP11A1 |  | SREBF1 |
|  | NPC1 |  | PRKACA |
|  | MT-ND4L |  | LDHA |
|  | MYD88 |  | PDGFB |
|  | PROP1 |  | F8 |
|  | LDLR |  | CHRM3 |
|  | CAT |  | FGA |
|  | FCGR3A |  | PTH |
|  | EDNRA |  | DDC |
|  | KDM5C |  | HTR2A |
|  | CCND2 |  | PSEN2 |
|  | NOS2 |  | CSNK2B |
|  | SPAST |  | OXT |
|  | CGA |  | HMGB1 |
|  | TAC3 |  | PTH1R |
|  | NPY |  | ACAN |
|  | SST |  | AHSG |
|  | VDR |  | DSG1 |
|  | JUN |  | CD163 |
|  | TFRC |  | HP |
|  | ESR2 |  | TSHR |
|  | GCDH |  | NPPB |
|  | CD59 |  | VTN |
|  | ITGB2 |  | TPO |
|  | PPP2R1A |  | LTF |
|  | IRF3 |  | PLAT |
|  | IGFBP3 |  | IL7 |
|  | DPP4 |  | PLAU |
|  | HCRT |  | TUBB |
|  | MTTP |  | MYO6 |
|  | CRYAB |  | DUOX2 |
|  | MMP1 |  | PCCB |
|  | AFP |  | UCP2 |
|  | TG |  | POU1F1 |
|  | AVPR2 |  | RPL10 |
|  | MME |  | ANGPT2 |
|  | LHX3 |  | ADAM17 |
|  | HMGCR |  | ADRB2 |
|  | VIP |  | CTSK |
|  | PGK1 |  | IRF1 |
|  | EPHX1 |  | IGFBP1 |
|  | FOS |  | ACP5 |
|  | TPM3 |  | TPM1 |
|  | EPO |  | SOD2 |
|  | AGTR1 |  | TPI1 |
|  | CLU |  | INSL3 |
|  | CLCN5 |  | AHCY |
|  | CALR |  | LHB |
|  | FSHB |  | FGF7 |
|  | CASP1 |  | CYB5A |
|  | MC2R |  | GSTP1 |
|  | GCG |  | ITGB1 |
|  | SPP1 |  | CTSB |
|  | SRSF2 |  | GPI |
|  | GGT1 |  | ATP1A1 |
|  | OAS1 |  | TGFA |
|  | FST |  | ADM |
|  | CALCA |  | HBEGF |
|  | RHO |  | HYAL1 |
|  | BGLAP |  | IL21 |
|  | GPX4 |  | POU5F1 |
|  | HSP90AA1 |  | ITGB6 |
|  | STAT5A |  | MC4R |
|  | MMP14 |  | APOC3 |
|  | GLUL |  | GHRHR |
|  | PECAM1 |  | TXN |
|  | BEST1 |  | SPEF2 |
|  | ARG1 |  | SPINK1 |
|  | BCL2L1 |  | KCNN3 |
|  | MDH2 |  | TNC |
|  | VIM |  | ANPEP |
|  | CHGA |  | FUT8 |
|  | SLC9A1 |  | ADCYAP1 |
|  | SELE |  | LIPE |
|  | TLR9 |  | GAL |
|  | SLC11A1 |  | AQP1 |
|  | CTSL |  | SCT |
|  | MB |  | LCAT |
|  | VCL |  | THRA |
|  | CCN2 |  | CCK |
|  | ANKRD1 |  | ANGPT1 |
|  | C5 |  | IGFBP2 |
|  | MSN |  | SERPINA7 |
|  | SYK |  | TRH |
|  | NAXE |  | GRP |
|  | NTRK3 |  | ANG |
|  | ALDH2 |  | MSMO1 |
|  | BCHE |  | MX1 |
|  | SLPI |  | OPTN |
|  | FOXO1 |  | TYROBP |
|  | PCK1 |  | ACADL |
|  | PRKACB |  | F9 |
|  | SPARC |  | MSMB |
|  | MYF6 |  | RDX |
|  | IL15 |  | GAST |
|  | F12 |  | NPPC |
|  | SAT1 |  | IL12RB2 |
|  | OPRM1 |  | TSHB |
|  | SAG |  | AK1 |
|  | CCL4 |  | NTN1 |
|  | SCG5 |  | PNLIP |
|  | SCARB1 |  | ZP3 |
|  | MBP |  | EPHX2 |
|  | SLC4A4 |  | FABP4 |
|  | DCN |  | GUCY2C |
|  | CFD |  | PPBP |
|  | ADRA2A |  | INHBA |
|  | FUT2 |  | CDH5 |
|  | GHRH |  | DMBT1 |
|  | SLC2A4 |  | GPX1 |
|  | SI |  | MSTN |
|  | BECN1 |  | SIGLEC1 |
|  | PF4 |  | PRKAA2 |
|  | UBC |  | CNTF |
|  | PYY |  | ANXA1 |
|  | INHA |  | PGRMC1 |
|  | CDKN3 |  | PRKAG3 |
|  | HPD |  | PRLR |
|  | IGFBP4 |  | FGF1 |
|  | GAD2 |  | IAPP |
|  | PDYN |  | CFL1 |
|  | RPSA |  | TGFBR3 |
|  | PFKM |  | CNP |
|  | MC1R |  | IYD |
|  | ATP2A2 |  | FAAH |
|  | SLC5A1 |  | FTCD |
|  | CAST |  | SSTR2 |
|  | ARAF |  | FABP2 |
|  | UCP3 |  | CD1A |
|  | ENTPD1 |  | MYOG |
|  | GYPA |  | ZP2 |
|  | PROC |  | ABCG2 |
|  | ANXA2 |  | CDC25C |
|  | ACP1 |  | AMBP |
|  | DNASE1 |  | CTSH |
|  | KLRK1 |  | HTR4 |
|  | LGALS1 |  | BHMT |
|  | CYP2E1 |  | SOCS2 |
|  | CS |  | TGFBI |
|  | DNASE2 |  | DUOX1 |
|  | BIRC5 |  | RPL35 |
|  | CD47 |  | CSNK1A1 |
|  | TXNIP |  | ITIH4 |
|  | AQP3 |  | FABP1 |
|  | DDX39B |  | ZP4 |
|  | GCGR |  | PCSK2 |
|  | ACR |  | TFF3 |
|  | OPRD1 |  | MAN2B2 |
|  | IGFBP5 |  | ALOX15 |
|  | LGALS4 |  | ZFY |
|  | HSP90B1 |  | PRDX2 |
|  | FYN |  | FOLH1 |
|  | CALCRL |  | PTAFR |
|  | IBSP |  | GUCA2A |
|  | NTF3 |  | BID |
|  | ADAM10 |  | CALCR |
|  | DIO3 |  | S100A6 |
|  | AKR1B1 |  | RNASE2 |
|  | ADRB1 |  | PLIN2 |
|  | GHSR |  | GUCA2B |
|  | LCN1 |  | OPRL1 |
|  | CHGB |  | PDXK |
|  | HSPA1A |  | HTR2B |
|  | GIP |  | NLN |
|  | AOC1 |  | PGD |
|  | AKR1A1 |  | RSAD2 |
|  | PRKAA1 |  | RAMP1 |
|  | PNOC |  | NMBR |
|  | IMPA1 |  | LDHC |
|  | CLCA1 |  | PTN |
|  | AGRP |  | PPY |
|  | IVL |  | NPY2R |
|  | TUBA1B |  | MFGE8 |
|  | DIO2 |  | NPY1R |
|  | WFDC2 |  | BDKRB2 |
|  | NMB |  | MMP20 |
|  | SLC22A2 |  | CCRL2 |
|  | CSN1S1 |  | RNASE1 |
|  | DRD1 |  | FDX1 |
|  | PCDH11X |  | ZFX |
|  | PPP2R1B |  | ANXA4 |
|  | CHRM2 |  | CLPS |
|  | HTR1B |  | PTGER3 |
|  | PLA2G1B |  | ODF1 |
|  | AZU1 |  | ENAM |
|  | MCHR1 |  | RNASE4 |
|  | PSMC5 |  | FMOD |
|  | S100A10 |  | RPL29 |
|  | SORBS2 |  | CA3 |
|  | NPY5R |  | HYAL3 |
|  | CCS |  | DGKA |
|  | CYSLTR2 |  | PTGR1 |
|  | MYL6 |  | NNAT |
|  | TNP2 |  | UCHL3 |
|  | LALBA |  | PLCD4 |
|  | ZAN |  | MCIDAS |
|  | PREP |  | DEFB1 |
|  | XPNPEP2 |  | NDUFB6 |
|  | LAP3 |  | COX5B |
|  | CCL25 |  | COX7A1 |
|  | UBE2D2 |  | CYBA |
|  | ALDH9A1 |  | CYSLTR1 |
|  | SLC22A6 |  | HPRT1 |
|  | CSN2 |  | RAB3A |
|  | TXNRD1 |  | PPARGC1A |
|  | ENPEP |  | IFNAR1 |
|  | POU2F1 |  | IL6R |
|  | HOPX |  | TNP1 |
|  | TMPRSS15 |  | IL12B |
|  | GPR4 |  | HEXB |
|  | PPP2CB |  | JAK2 |
|  | DPEP1 |  | IL12A |
|  | HCRTR2 |  | KCNQ1 |
|  | SGMS1 |  | BLOC1S3 |
|  | NMU |  | FCER1G |
|  | SLC28A1 |  | CD3E |
|  | DPT |  | TRAF6 |
|  | RLN3 |  | AKIRIN2 |
|  | LMCD1 |  | ALOX5AP |
|  | S100G |  | P2RY2 |
|  | RGS16 |  | DDOST |
|  | CBR1 |  | UBA52 |
|  | MYOZ1 |  | MEF2C |
|  | DDO |  | DSTN |
|  | GUK1 |  | ASIP |
|  | FXYD1 |  | RPL4 |
|  | TSC22D3 |  | S100A11 |
|  | LEPROT |  | PHPT1 |
