# Supplementary material for: Antiviral effects and mechanism of Ma-Xing-Shi-Gan-San on porcine reproductive and respiratory syndrome virus
Source: Front Microbiol. 2025 Apr 29;16:1539094. doi: 10.3389/fmicb.2025.1539094 (PMC12069324; doi:10.3389/fmicb.2025.1539094)
Supplement: Supplementary file 4 [file Table_4.DOCX]

Table S4 The intersection of MXSGS and PRRSV targets

| **Serial number** | **The comment target** | **Serial number** | **The comment target** |
| --- | --- | --- | --- |
|  | HTR2B |  | DRD1 |
|  | CYP19A1 |  | ADRB2 |
|  | ESR1 |  | ADRB1 |
|  | ESR2 |  | HTR2A |
|  | NR3C1 |  | KCNN3 |
|  | IGF1R |  | ADRA2A |
|  | ALOX15 |  | BCHE |
|  | SLC22A2 |  | JUN |
|  | CFTR |  | HCRTR2 |
|  | OPRM1 |  | PIK3CG |
|  | CYP17A1 |  | SERPINE1 |
|  | FYN |  | KCNH2 |
|  | MET |  | JAK2 |
|  | CBR1 |  | CDC42 |
|  | OPRD1 |  | ADAM10 |
|  | PSEN2 |  | HSP90B1 |
|  | TTR |  | AVPR2 |
|  | ALDH2 |  | F2 |
|  | AKR1B1 |  | PLG |
|  | DHFR |  | PRKAA1 |
|  | HSP90AA1 |  | KCNMA1 |
|  | MMP1 |  | PTAFR |
|  | GCGR |  | PRKACA |
|  | TLR9 |  | RYR1 |
|  | ADAM17 |  | PLCD4 |
|  | MMP14 |  | SPARC |
|  | EPHX2 |  | ALB |
|  | COMT |  | F9 |
|  | TGFBR1 |  | TF |
|  | PLA2G1B |  | LTF |
|  | NPY5R |  | PROC |
|  | IDH1 |  | CTSK |
|  | VCP |  | SLC5A1 |
|  | DPP4 |  | SLC4A4 |
|  | ALOX5AP |  | SLC2A2 |
|  | ABCG2 |  | ARG1 |
|  | NOS2 |  | SYK |
|  | SNCA |  | AKR1A1 |
|  | BCL2L1 |  | PREP |
|  | PGD |  | FAAH |
|  | PLAU |  | OXTR |
|  | CA3 |  | PPARG |
|  | IGFBP3 |  | EDNRA |
|  | PLAT |  | CTSB |
|  | THRA |  | PTGER3 |
|  | SLC9A1 |  | VEGFA |
|  | MYLK |  | KCNJ5 |
|  | CTSD |  | OPRL1 |
|  | IL2 |  | PRKACB |
|  | HSPA1A |  | HTR4 |
|  | CDC25C |  | ACE |
|  | CTSL |  | CALCRL |
|  | TNF |  | SSTR2 |
|  | IGFBP4 |  | CASR |
|  | IGFBP5 |  | CYSLTR1 |
|  | IGFBP2 |  | CHRM3 |
|  | IGFBP1 |  | NOS3 |
|  | KDM5C |  | CASP3 |
|  | EPHX1 |  | NR5A1 |
|  | TGFB1 |  | GNRHR |
|  | PGK1 |  | ACP1 |
|  | GSTP1 |  | CYSLTR2 |
|  | BDKRB2 |  | CALCA |
|  | MME |  | HMGCR |
|  | AGTR1 |  | CHRM2 |
|  | TLR4 |  | CSNK1A1 |
|  | EDNRB |  | CFD |
|  | LDLR |  | ANPEP |
|  | STAT3 |  | NPY1R |
|  | HTR1B |  | BIRC5 |
